# Supplementary material for: Relative exchangeable copper: A highly specific and sensitive biomarker for Wilson disease diagnosis
Source: JHEP Rep. 2025 Jul 31;7(10):101537. doi: 10.1016/j.jhepr.2025.101537 (PMC12446559; doi:10.1016/j.jhepr.2025.101537)
Supplement: Multimedia component 1 [file mmc1.pdf]

Relative exchangeable copper: A highly specific and sensitive biomarker for Wilson disease diagnosis

Nouzha Djebrani-Oussedik, Clément Desjardins, Mickaël Alexandre Obadia, Djamila Rahli, Corinne Collet, France Woimant, Joël Poupon, Dominique Debray, Aurélia Poujois

Table of contents

Supplementary table legends……………………………………………………………2

Table S1………………………………………………………………………………3

Table S2……………………………………………………………………………...27

Table S3……………………………………………………………………………...29

Table S4………………………………………………………………………………31

Table S5……………………………………………………………………………...33

Supplementary table legends:

Table S1. Genetic data of Wilson's Disease (WD) patients and Heterozygous carriers (HTZ).

Table S2. Results of copper metabolism assessment in controls, with comparisons by sex

Serum copper and CuEXC conversion: µmol/L × 6.3546 = µg/dL. F: female; M: male.

Table S3. Results of copper metabolism assessment in controls, with comparisons across age groups.

Serum copper and CuEXC conversion: µmol/L × 6.3546 = µg/dL. F: female; M: male.

Table S4. Main clinical, biological and genetic features associated with false negative and false positive REC results. F: Female; M: Male; Total Cu: Total serum copper; CuEXC: Exchangeable copper; REC: Relative exchangeable copper; Cp: Ceruloplasmin; 24 hr-UCE: 24-hour urinary copper excrection; AST: Aspartate aminotransferase; ALT: Alanine aminotransferase; PT: Prothrombin rate; KFR: Kayser Fleischer Ring; CDG: Congenital Disorder of Glycosylation.

Reference ranges were determined across all control samples

[1] Reference range: 8.5-28.2 µmol/L (24-179.2 µg/dL)

[2] Reference range: 0.50-1.38 µmol/L (3.2-8.8 µg/dL)

[3] Reference range: 2.6-9.5%

[4] Reference range: 0.17-0.4 g/L

[5] Reference range: 0.03-1.679 µmol/24h

Table S5. Comparison of total copper, exchangeable copper (CuEXC), and relative exchangeable copper (REC) levels in controls and heterozygous carriers (HTZ) across two time periods (2009–2014 vs. after 2015).

Table S1. Genetic data of (A.) Wilson Disease (WD) patients and (B.) Heterozygous carriers (HTZ)

A. **Variants in the 204 WD patients**

ALLELE 1	ALLELE 2
Exon 2 : c.676C>T, p.Arg226Trp<br><br>Exon 8: c.2297C>G, p.Thr766Arg	Exon 2 : c.676C>T, p.Arg226Trp<br><br>Exon 8: c.2297C>G, p.Thr766Arg
Exon 2 : c.676C>T, p.Arg226Trp<br><br>Exon 8: c.2297C>G, p.Thr766Arg	Exon 2 : c.676C>T, p.Arg226Trp<br><br>Exon 8: c.2297C>G, p.Thr766Arg
Exon 3 : c.804dupT, p.Leu269Serfs*14<br><br>Exon 21: c.4135C>T, p.Pro1379Ser	Exon 18 : c.3859G>A, p.Gly1287Ser
Exon 2 : c.676C>T, p.Arg226Trp<br><br>Exon 16: c.3556G>A, p.Gly1186Ser	Exon 8 : c.2297C>G, p.Thr766Arg
Exon 1 : c.19_20delCA, p.Gln7Aspfs*14<br><br>Exon 18: c.3809A>G, p.Asn1270Ser	Exon 14 : c.3207C>A, p.His1069Gln
Exon 6 : c.1877G>C, p.Gly626Ala	Exon 6 : c.1877G>C, p.Gly626Ala<br><br>Exon 16 : c.3443T>C, p.Ile1148Thr
Intron 2 : c.1285+1G>A, p.?	Exon 8: c.2332>T, p.Arg778Trp<br><br>Exon 16 c.3451C>T, p.Arg1151Cys
Exon 2 : c.804dupT, p.Lys269Ter<br><br>Exon 21 : c.4135C>T, p.Pro1379Ser	Exon 18 : c.3859G>A, p.Gly1287Ser
Exon 2 : c.676C>T, p.Arg226Trp	Exon 6 : c.1877G>C, p.Gly626Ala<br><br>Exon 8 : c.2297C>G, p.Thr766Arg
Intron 18 : c.3904-2A>G, p.Val1302Glyfs*8	Exons 1-7: c.52-?_1285+?del
Exon 13 : c.3007G>A, p.Ala1003Thr	Exon14 : c.3106G>A, p.Val1036Ile
Exon 8 : c.2293G>A, p.Asp765Asn	Exon 8 : c.2293G>A, p.Asp765Asn
Exon 2 : c.122A>G, p.Asn41Ser	Exon 15 : c.3402del, p.Ala1135Glnfs*13

**Exon 17 : c.3694 A>C, p.Thr1232Pro**	**Exon 17 : c.3694 A>C, p.Thr1232Pro**
**Exon 6 : c.1924G>C, p.Asp642His**	**Exon 8 : c.2131G>C, p.Gly711Arg**
**Exon 10 : c2558A>G, p.Asp853Gly**	**Exon 14 : c.3115G>T, p.Val1039Phe**
**Exon 10 : c2558A>G, p.Asp853Gly**	**Exon 14 : c.3115G>T, p.Val1039Phe**
**Exon 14 : c.3182G>A, p.Gly1061Glu**	**Exon 19 : c.3971A>G, p.Asn1324Ser**
**Exon 14 : c.3182G>A, p.Gly1061Glu**	**Exon 19 : c.3971A>G, p.Asn1324Ser**
**Exon 16 : c.3509delC, p.Thr1170Lysfs*5**	**Exon 18 : c.3843dupT, p.Val1282Cysfs*22**
**Intron 5 : c.1708-1G>A, p.?**	**Intron 5 : c.1708-1G>A, p.?**
**Intron 5 : c.1708-1G>A, p.?**	**Intron 5 : c.1708-1G>A, p.?**
**Intron 5 : c.1708-1G>A, p.?**	**Intron 5 : c.1708-1G>A, p.?**
**Intron 1 : c.51+4A>T, p.?**	**Exon 18 : c.3796G>A, p.Gly1266Arg**
**Exon 13 : c.2963G>T, p.Gly988Val**	**Exon 18 : c.3809A>G, p.Asn1270Ser**
**Exon 10 : c.2507G>A, p.Gly836Glu**	**Exon 10 : c.2507G>A, p.Gly836Glu**
**Exon 15 : c.3293C>G, p.Pro1098Arg**	**Exon 15 : c.3293C>G, p.Pro1098Arg**
**Exon 15 : c.3293C>G, p.Pro1098Arg**	**Exon 15 : c.3293C>G, p.Pro1098Arg**
**Intron 1 : c.51+4A>T, p.?**	**Intron 1 : c.51+4A>T, p.?**
**Intron 1 : c.51+4A>T, p.?**	**Intron 1 : c.51+4A>T, p.?**
**Intron 1 : c.51+4A>T, p.?**	**Intron 1 : c.51+4A>T, p.?**
**Intron 4 : c.1708-1G>A, p.?**	**Exon 17 : c.3620A>G, p.His1207Arg**
**Intron 8 : c.2122-8T>G, p.?**	**Exon 13 : c.2297C>T, p.Thr766Met**
**Exon 7 : c.1995G>A, p.Met665Ile**	**Exon 16 : c.3445G>A, p.Gly1149Arg**
**Exon 16 : c.3505A>G, p.Met1169Val**	**Exon 17 : c.3659C>T, p.Thr1220Met**
**Exon 20 : c.4112T>C, p.Leu1371Pro**	**Exon 20 : c.4112T>C, p.Leu1371Pro**
**Exon 14 : c.3207C>A, p.His1069Gln**	**Exon 14 : c.3207C>A, p.His1069Gln**
**Exon 8 : c.2128G>A, p.Gly710Ser**	**Exon 14 : c.3207C>A, p.His1069Gln**

Exon 14 : c.3207C>A, p.His1069Gln	Intron 15 : c.3556+1G>T, p.Arg1186Glufs*10
Exon 14 : c.3207C>A, p.His1069Gln	Intron 15 : c.3556+1G>T, p.Arg1186Glufs*10
Exon 10 : c.2507G>A, p.Gly836Glu	Unidentified
Exon 8 : c.2333G>A, p.Arg778Gln	Exon 14: c.3207C>A, p.His1069Gln
Exon 13 : c.3059A>G, p.Lys1020Arg	Exon 13: c.3059A>G, p.Lys1020Arg
Exon 4 : c.1543+1_1544-1_1707+1_1708-1del, p.?	Exon 2 : c.388C>T, p.Arg130Cys
Exon 14 : c.3207C>A, p.His1069Gln	Exon 4 : deletion
Exon 8 : c.2297C>G, p.Thr766Arg	Exon 8 : c.2332C>T, p.Arg778Trp
Exon 18 : c.3799G>A, p.Asp1267Asn	Intron 4 : c.1708-34G>A, p.?
Intron 10 : c.1869+2T>C, p.Ser623Alafs*23	Exon 10 : c.2575G>A, p.Gly859Arg
Exon 20 : c.4092_4093del, p.Ser1365Cysfs*12	Intron 20 : c.4125-1G>T, p.?
Exon 20 : c.4092_4093del, p.Ser1365Cysfs*12	Intron 20 : c.4125-1G>T, p.?
Intron 4 : c.708-1G>A, p.?	Exon 6 : c.1934T>G, p.Met645Arg
Intron 4 : c.708-1G>A, p.?	Intron 4 : c.708-1G>A, p.?
Exon 2 : c.347T>C, p.Ile116Thr	Exon 13 : c.2930C>T, p.Thr977Met
Exon 14 : c.3207C>A, p.His1069Gln	Exon 15 : c.3263T>A, p.Leu1088*
Intron 1 : c.51+4A>T, p.?	Exon 17: c.3694A>C, p.Thr1232Pro
Exon 12 : c.2795C>A, p.Ser932Ter	Exon 12: c.2795C>A, p.Ser932Ter
Exon 12 : c.2795C>A, p.Ser932Ter	Exon 12 : c.2795C>A, p.Ser932Ter
Intron 4 : c.1707+2dupT	Intron 4 : c.1707+2dupT
Intron 4 : c.1707+2dupT	Intron 4 : c.1707+2dupT
Exon 8 : c.2297C>G, p.Thr766Arg	Exon 14 : c.3207C>A, p.His1069Gln
Exon 14 : c.3207C>A, p.His1069Gln	Exon 14 : c.3207C>A, p.His1069Gln

**Exon 8 : c.2128G>A, p.Gly710Ser**	**Exon 16 : c.3505A>G, p.Met1169Val**
**Exon 8 : c.2303C>T, p.Pro768Leu**	**Exon 20 : c.4092_4093del, p.Ser1365Cysfs*12**
**Exon 14 : c.3207C>A, p.His1069Gln**	**Exon 14 : c.3207C>A, p.His1069Gln**
**Exon 16 : c.3443T>C, p.Ile1148Thr**	**Exon 18 : c.3818C>T, p.Pro1273Leu**
**Exon 12 : c.2804C>T, p.Thr935Met**	**Exon 12 : c.2804C>T, p.Thr935Met**
**Exon 12 : c.2804C>T, p.Thr935Met**	**Exon 12 : c.2804C>T, p.Thr935Met**
**Exon 12 : c.2804C>T, p.Thr935Met**	**Exon 12 : c.2804C>T, p.Thr935Met**
**Exon 4 : c.1705_1707-10del, p.Thr569del**	**Exon 5 : c.1745_1746del, p.Ile582Argfs*25**
**Exon 10 : c.2532delA, pVal845 Serfs*28**	**Exon 18 : c.3821C>A, p.Ala1274Asp**
**Exon 10 : c.2532delA, pVal845 Serfs*28**	**Exon 18 : c.3821C>A, p.Ala1274Asp**
**Exon 10 : c.2532delA, pVal845 Serfs*28**	**Exon 18 : c.3821C>A, p.Ala1274Asp**
**Exon 14 : c.3207C>A, p.His1069Gln**	**Exon 17 : c.3646G>A, p.Val1216Met**
**Exon 2 : c.331C>T, p.Gln111***	**Exon 7 : c.1995G>A, p.Met665Ile**
**Exon 8 : c.2305A>G, p.Met769Val**	**Exon 11 : c.2605G>T, p.Gly869Ter**
**Exon 8 : c.2304dup, p.Met769Hisfs*26**	**Exon 8 : c.2304dup, p.Met769Hisfs*26**
**Exon 6 : c.1774A>T, p.Ile592Phe**	**Exon 4 : c.1145C>G, p.Ser382Cys**
**Exon 14 : c.3207C>A, p.His1069Gln**	**Exon 14 : c.3207C>A, p.His1069Gln**
**Exon 14 : c.3083_3085delinsG>C, p.Lys1028Serfs*40**	**Exon 16 : c.3445G>C, p.Gly1149Arg**
**Exon 8 : c.2128G>A, p.Gly710Ser**	**Intron 19 : c.4124+6T>C, p.?**
**Exon 8 : c.2336G>A, p.Trp779Ter**	**Exon 14: c.3207C>A, p.His1069Gln**
**Exon 19 : c.3955C>T, p.Arg1319Ter**	**Exon 19 : c.3955C>T, p.Arg1319Ter**
**Intron 4 : c.-362C>T, p.?**	**5'UTR / prom: c.-362C>T, p.?**
**Exon 3 : c.958_961delinsAGTT, p.Pro320Serfs*6**	**Exon 3 : c.958_961delinsAGTT, p.Pro320Serfs*6**

Intron 13 : c.3061-12T>A, p.?	Unidentified
Exon 8 : c.2128G>A, p.Gly710Ser	Exon 8 : c.2304dupC, p.Met769HisfsX26
Exon 5 : c.1708-1G>A, p.Glu570Alafs*13	Exon 5 : c.1708-1G>A, p.Glu570Alafs*13
Exon 5 : c.122del, p.Asn41fs	Intron 12 : c.2576-2A>G, p.Lys859Argfs*10
Intron 1 : c.51+4A>T, p.?	Exon 8 : c.2128G>A, p.Gly710Ser
Exon 6 : c.1934T>G, p.Met645Arg	Exon 18 : c.3809A>G, p.Asn1270Ser
Exon 2 : c.778dupC, p.Gln260ProfsX10	Intron 10 : c.1869+5_1869+8del, p.?
Exon 15 : c.3263T>A, p.Leu1088*	Intron 15 : c.3412+2T>C, p.?
Exon 11 : c.2605G>A, p.Gly869Arg	Intron 18 : c.3412+2T>C, p.Leu1138Profs*19
Exon 8 : c.2304dupC, p.Met769Hisfs*26	Exon 1 : c.3190G>A, p.Glu1064Lys
Exon 18 : c.3842G>A, p.Gly1281Asp	Intron 17 : c.3699+280A>G, p.?
Exon 7 : c.1995G>A, p.Met665IIe	Intron1 : c.51+4A>T, p.?
Exon 2 : c.865C>T, p.Gln289X	Exon 8 : c.232C>G, p.Arg778Gly
Exon 13 : c.3059A>G, p.Lys1020Arg	Exon 13 : c.3059A>G, p.Lys1020Arg
Exon 13 : c.3059A>G, p.Lys1020Arg	Exon 13 : c.3059A>G, p.Lys1020Arg
Exon 13 : c.3059A>G, p.Lys1020Arg	Exon 13 : c.3059A>G, p.Lys1020Arg
Exon 19 : c.3955C>T, p.Arg1319*	Exon 19 : c.3955C>T, p.Arg1319*
Intron 9 : c.2448-41G>A, p.?	Unidentified
Exon 13 : c.2930C>T, p.Thr977Met	Intron 13 : c.3061-1G>A, p.Gly1021Glufs*16
Intron 1 : c.51+4A>T, p.?	Intron 5 : c.1708-1G>C, p.?
Exon 2 : c.122A>G, p.Asn41Ser	Exon 18 : c.3743A>C, p.Lys1248Thr
Exon 2 : c.122A>G, p.Asn41Ser	Exon 18 : c.3743A>C, p.Lys1248Thr
Exon 18 : c.3809A>G, p.Asn1270Ser	Exon 2: insertion ? C.?, p.?
Exon 17 : c.3694 A>C, p.Thr1232Pro	Exon 17 : c.3694A>C, p.Trp1232Pro
Exon 1 : c.51+5G>A, p.?	Exon 10 : c.2549C>A, p.Thr850Asn

**Exon 19 : c.3971A>G, p.Asn1324Ser**	**Exon 20 : c.4040delG, p.Gly1347AlafsTer46**
**Exon 13 : c.3007G>A, p.Ala1003Thr**	**Exon 14 : c.3207C>A, p.His1069Gln**
**Exon 18 : c.3895C>T, p.Leu1299Phe**	**Exon 18 : c.3895C>T, p.Leu1299Phe**
**Exon 14 : c.3188C>T, p.Ala1063Val**	**Exon 18 : c.3743A>C, p.Lys1248Thr**
**Intron 1 : c.51+4A>T, p.?**	**Exon 8 : c.2237T>A, p.Val746Asp**
**Intron 1 : c.51+4A>T, p.?**	**Exon 8 : c.2237T>A, p.Val746Asp**
**Exon 12 : c.2804C>T, p.Thr935Met**	**Exon 12: c.2804C>T, p.Thr935Met**
**Exon 2 : c.1136del, p.Gly379Alafs*2**	**Exon 8 : c.2333G>T, p.Arg778Leu**
**Exon 11 : c.2621C>T, p.Ala874Val**	**Exon 14 : c.3182G>A, p.Gly1061Glu**
**Exon 14 : c.3207C>A, p.His1069Gln**	**Exon 15 : c.3402del, p.Ala1135GlnfsTer13**
**Exon 14 : c.3207C>A, p.His1069Gln**	**Exon 18 : c.3842G>A, p.Gly1281Asp**
**Exon 2 : c.505C>T, p.Gln169***	**Exon 13 : c.2936T>G, p.Leu979Arg**
**Exon 6 : c.1934T>G p.Met645Arg**	**Exon 13 : c.2911G>C, p.Ala971Pro**
**Exon 14 : c.3207C>A, p.His1069Gln**	**Exon 16 : c.3547G>A, p.Ala1183Thr**
**Exon 21 : c.4144G>T p.Glu1382***	**Exon 21 : c.4144G>T, p.Glu1382***
**Exon 7 : c.2009_2015del, p.Tyr670_Met671delinsTer**	**Exon 13 : c.3035G>T, p.Gly1012Val**
**Exon 14 : c.3207C>A, p.His1069Gln**	**Unidentified**
**Exon 13 : c.2930C>T, p.Thr977Met**	**Exon 13 : c.2936T>A, p.Leu979Gln**
**Intron 10 : c.1869+5_1869+8delGTAA, p.?**	**Exon 19 : c.3914T>C, p.Leu1305Pro**
**Exon 16 : c.3461T>C, p.Leu1154Pro**	**Exon 18 : c.3842G>A, p.Gly1281Asp**
**Exon 13 : c.3053C>T; p.Ala1018Val**	**Exon 17 : c.3617_3618insA, p.His1207Alafs*52**
**No variant detected in 2012 (Sanger and MLPA)**	**No variant detected in 2012 (Sanger and MLPA)**
**Unidentified ~~No variant detected in 2012 (Sanger and MLPA)~~**	**Unidentified ~~No variant detected in 2012 (Sanger and MLPA)~~**

**Exon 13 : c.3059A>G, p.Lys1020Arg**	**Exon 13 : c.3059A>G, p.Lys1020Arg**
**Exon 7 : c.3659C<T, p.Thr1220Met**	**Exon 14 : c.3083-3085delinsGT, p.Lys1028Serfs*40**
**Exon 14 : c.3098C>T, p.Thr1033Ile**	**Exon 14 : c.3098C>T, p.Thr1033Ile**
**Exon 8 : c.2336G>A, p.Trp779Ter**	**Exon 14: c.3063A>G, p.Ile1021Met**
**Exon 13 : c.2912C>T, p.Ala971Val**	**Intron 16 : c.3556+1G>T, p.?**
**Exon 10 : c2519C>T, p.Pro840Leu**	**Exon 14: c.3104G>A, p.Gly1035Asp**
**Exon 13 : c.3059A>G, p.Lys1020Arg**	**Exon 13 : c.3059A>G, p.Lys1020Arg**
**Exon 8 : c.2304dupC, p.Met769Hisfs*26**	**Exon 8 : c.2304dupC, p.Met769Hisfs*26**
**Exon 2 : c.813C>A, p.Cys271***	**Exon 8 : c.2145C>T, p.Tyr715Tyr**
**Exon 14 : c.3207C>A, p.His1069Gln**	**Exon 17 : c.3694A>C, p.Thr1232Pro**
**Exon 10 : c.2549C>T, p.Thr850Ile**	**Exon 18 : c.3818C>A, p.Pro1273Gln**
**Exon 10 : c.2549C>T, p.Thr850Ile**	**Exon 18 : c.3818C>A, p.Pro1273Gln**
**Exon 2 : c.314C>A, p.Ser105***	**Exon 5 : c.1846C>T, p.Arg616Trp**
**Exon 6 : c.1934T>G, p.Met645Arg**	**Exon 6 : c.1934T>G, p.Met645Arg**
**Exon 6 : c.1934T>G, p.Met645Arg**	**Exon 6 : c.1934T>G, p.Met645Arg**
**Intron 1 : c.51+4A>T, p.?**	**Intron 1 : c.51+4A>T, p.?**
**Exon 14 : c.3207C>A, p.His1069Gln**	**Exon 14 : c.3207C>A, p.His1069Gln**
**Exon 10 : c.2532del:p.Val845Serfs*28**	**Exon 10 : c.2532del:p.Val845Serfs*28**
**Exon 8 : c.2292C>T, p.Phe764Phe**	**Exon 8 : c.2292C>T, p.Phe764Phe**
**Exon 2 : c.213_214delAT, p.Val73Glufs*4**	**Unidentified**
**Exon 14 : c.3207C>A, p.His1069Gln**	**Exon 18 : c.3809A>G, p.Asn1270Ser**
**Exon 4 : c.1705_1707+10del13 p.Thr569del**	**Exon 4 : c.1705_1707+10del13, p.Thr569del**
**Exon 14 : c.3207C>A, p.His1069Gln**	**Exon 14 : c.3207C>A, p.His1069Gln**
**Exon 2 : c.331C>T, p.Gln111Ter**	**Exon 19 : c.3914T>C, p.Leu1305Pro**

**Exon 6 : c.1934T>G, p.Met645Arg**	**Unidentified**
**Exon 2 : c.670dup, p.Ile224Asnfs*2**	**Exon 7 : c.2046G>C, p.Met682Ile**
**Exon 14 : c.3207C>A, p.His1069Gln**	**Exon 19 : c.3914T>C, p.Leu1305Pro**
**Exon 8 : c.2332C>T, p.Arg778Trp**	**Exon 20 : c.4049T>G, p.Leu1350Arg**
**Exon 19 : c.4021G>A, p.Gly1341Ser**	**Unidentified**
**Exon 13 : c.2971A>G, p.Thr991Ala**	**Exon 18 : c.3872C>T, p.Ala1291Val**
**Exon 8 : c.2128G>A, p.Gly710Ser**	**Exon 8 : c.2305A>G, p.Met769Val**
**Exon 8 : c.2304dupC, p.Met769Hisfs*26**	**Exon 20 : c.4124G>C, p.Cys1375Ser**
**Exon 8 : c.2304dupC, p.Met769Hisfs*26**	**Exon 14 : c.3207C>A, p.His1069Gln**
**Exon 4 : c.1625T>C p.Ile542Thr**	**Intron 11 : c.2731-1G>A, p.?**
**Exon 12 : c.2753A>G, p.Asp918Gly**	**Exon 16 : c.3556G>A, p.Gly1186Ser**
**Exon 12 : c.2753A>G, p.Asp918Gly**	**Exon 16 : c.3556G>A, p.Gly1186Ser**
**Exon 8 : c.2128G>A, p.Gly710Ser**	**Exon 8 : c.2304dupC, p.Met769Hisfs*26**
**Exon 14 : c.3061-12T>A p.(?)**	**Exon 19 : c.3955C>T, p.(Arg1319Ter)**
**Exon 2 : c.213_214delAT, p.Val73Glufs*4**	**Exon 7 : c.1995G>A, p.Met665Ile**
**Exon 2 : c.213_214delAT, p.Val73Glufs*4**	**Exon 7 : c.1995G>A, p.Met665Ile**
**Intron 1 : c.51+4A>T, p.?**	**Exon 3 : c.1512dup, p.Asn505Ter**
**Exon 2 : c.721C>T, p.Gln241***	**Exon 2: c.721C>T, p.Gln241***
**Exon 8 : c.2305A>G, p.Met769Val**	**Exon 20 : c.4051C>T, Gln1351***
**Exon 8 : c.2305A>G, p.Met769Val**	**Exon 20 : c.4051C>T, Gln1351***
**Intron 1 : c.51+4A>T, p.?**	**Exon 10 : c.2512A>G; p.Lys838glu**
**Intron 1 : c.51+4A>T, p.?**	**Exon 10 : c.2512A>G; p.Lys838glu**
**Intron 2 : c.1285+1G>A, p.?**	**Exon 10 : c.2501T>A, p.Val834Asp**
**Exon 5 : c.1774A>T, p.IIe592Phe**	**Exon 10: c.2525A>G, p.Asp842Gly**
**Intron 1 : c.51+4A>T, p.?**	**Intron 1 : c.51+4A>T, p.?**

**Intron 1 : c.51+4A>T, p.?**	**Intron 1 : c.51+4A>T, p.?**
**Exon 13 : c.3059A>G, p.Lys1020Arg**	**Exon 14 : c.3207C>A, p.His1069Gln**
**Intron 7 : c.2122-8T>G, p.?**	**Exon 14 : c.3207C>A, p.His1069Gln**
**Exon 8 : c.2324C>T, p.Ala775Val**	**Exon 19 : c.3992A>G,p.Tyr1331Cys**
**Exon 8 : c.2324C>T, p.Ala775Val**	**Exon 19 : c.3992A>G,p.Tyr1331Cys**
**Exon 16 : c.3551T>C, p.Ile1184Thr**	**Intron 12 : c.2866-1521G>A, p.?**
**Exon 12 : c.2752G>A, p.Asp918Asn**	**Exon 12 : c.2752G>A p.Asp918Asn**
**Intron 12 : c.2865+1G>A, p.?**	**Exon 14 : c.3083A>G, p.Lys1028Arg**
**Exon 8 : c.2123T>C, p.Leu708Pro**	**Exon 14 c.3207C>A, p.His1069Gln**
**Exon 3 : c.1512dupT, p.Asn505***	**Exon 9 : c.2383C>T, p,Leu795Phe**
**Intron 11 : c.2731-2A>G, p.?**	**Exon 17 : c.3688A>G, p.(Ile1230Val)**
**Intron 1 : c.51+4A>T, p.?**	**Exon 14 : c.3207C>A, p.His1069Gln**
**Exon 12 : c.2762G>A, p.Ser921Asn**	**Unidentified**
**Exon 14 : c.3207C>A, p.His1069Gln**	**Exon 14 : c.3207C>A, p.His1069Gln**
**Exon 6 : c.1877G>C p.Gly626Ala**	**Exon 18 : c.3842G>A p.Gly 1281Asp**
**Exon 14 : c.3191A>C, p.Glu1064Ala**	**Exon 17 : c.3694A>C, p.Thr1232Pro**
**Exon 14 : c.3191A>C, p.Glu1064Ala**	**Exon 17 : c.3694A>C, p.Thr1232Pro**
**Exon 16 : c.3426G>C, p.Gln1142His**	**Exon 16 : c.3443T>C, p.Ile1148Thr**
**Exon 8 : c.2333G>T, p.Arg778Leu**	**Exon 18 : c.3809A>G, p.Asn1270Ser**
**Exon 18 : c.3817C>T, p.Pro1273Leu**	**Exon 18 : c.3817C>T, p.Pro1273Leu**
**Exon 14 : c.3207C>A, p.His1069Gln**	**Exon 14 : c.3207C>A, p.His1069Gln**
**Exon 14 : c.3207C>A, p.His1069Gln**	**Exon 14 : c.3207C>A, p.His1069Gln**
**Exon 13 : c.2930C>T, p.Thr977Met**	**Exon 13 : c.2930C>T, p.Thr977Met**

B. Variants in the 359 Heterozygous carriers (HTZ)

ALLELE 1
**Exon 14 : c.3207C>A, p.His1069Gln**
**Exon 13 : c.3007G>A, p.Ala1003Thr and Exon14: c.3106G>A, p.Val1036Ile**
**Exon 8 : c.2293G>A, p.Asp765Asn**
**Exon 19 : c.4004G>A, p.Gly1335Glu**
**Exon 19 : c.4004G>A, p.Gly1335Glu**
**Exon 8 : c.2304dupC, p.Met769Hisfs*26**
**Exon 2 : c.1136delG, p.Gly379Alafs*2**
**Exon 8 : c.2304dupC, p.Met769Hisfs*26**
**Exon 14 : c.3071_3072delTG, p.Val1024Aspfs*3**
**Exon 6 : c.1924G>C, p.Asp642His**
**Exon 8 : c.2131G>C, p.Gly711Arg**
**Exon 8 : c.2131G>A, p.Gly711Arg**
**Exon 10 : c.2507G>A, p.Gly836Glu**
**Exon 13 : c.2963G>T, p.Gly988Val**
**Exon 13 : c.2963G>T, p.Gly988Val**
**Exon 18 : c.3809A>G, p.Asn1270Ser**
**Exon 2 : c.576_577insG, p.Gln193Alafs*11**
**Exon 10 : c.2532del, p.Val845SerfsX28**
**Exon 2 : c.1063C>T, p.Gln355***
**Intron 1 : c.51+4A>T, p.?**
**Intron 1 : c.51+4A>T, p.?**
**Intron 1 : c.51+4A>T, p.?**
**Intron 1 : c.51+4A>T, p.?**
**Exon 14 : c.3207C>A, p.His1069Gln**

**Exon 14 : c.3207C>A, p.His1069Gln**
**Exon 14 : c.3188C>T, p.Ala1063Val**
**Exon 10 : c.2549C>T, p.Thr850Ile**
**Exon 15 : c.3263T>A, p.Ile1088Ter**
**Exon 15 : c.3263T>A, p.Ile1088Ter**
**Exon 15 : c.3263T>A, p.Ile1088Ter**
**Exon 15 : c.3263T>A, p.Ile1088Ter**
**Exon 7 : c.2009_2015del, pTyr670_Met671delinsTer**
**Exon 2 : c.865C>T, p.Gln289***
**Exon 14 : c.3207C>A, p.His1069Gln**
**Exon 2 : c.213_214del, p.Val73GlufsTer4**
**Exon 2 : c.213_214del, p.Val73GlufsTer4**
**Intron 1 : c.51+4A>T, p.?**
**Exon 14 : c.3207C>A, p.His1069Gln**
**Exon 14 : c.3207C>A, p.His1069Gln**
**Exon 13 : c.3059A>G, p.Lys1020Arg**
**Exon 13 : c.3059A>G, p.Lys1020Arg**
**Exon 13 : c.3059A>G, p.Lys1020Arg**
**Exon 13 : c.3059A>G, p.Lys1020Arg**
**Exon 13 : c.3059A>G, p.Lys1020Arg**
**Intron 5 : c.1869+2T>C, p.?**
**Intron 5 : c.1869+2T>C, p.?**
**Exon 8 : c.2303C>T, p.Pro768Leu**
**Exon 3 : c.1512dupT, p.Asn505***
**Exon 14 : c.3207C>A, p.His1069Gln**

**Exon 14 : c.3207C>A, p.His1069Gln**
**Intron 1 : c.51+4A>T, p.?**
**Intron 1 : c.51+4A>T, p.?**
**Exon 20 : c.4092_4093delGT, p.Ser1365Lysfs*12**
**Exon 20 : c.4092_4093delGT, p.Ser1365Lysfs*12**
**Exon 20 : c.4092_4093delGT, p.Ser1365Lysfs*12**
**Exon 20 : c.4092_4093delGT, p.Ser1365Lysfs*12**
**Exon 18 : c.3809A>G, p.Asn1270Ser**
**Exon 18 : c.3809A>G, p.Asn1270Ser**
**Exon 18 : c.3809A>G, p.Asn1270Ser**
**Exon 19 : c.3955C>T, p.Arg1319***
**Exon 14 : c.3207C>A, p.His1069Gln**
**Exon 8 : c.2128G>A, p.Gly710Ser**
**Exon 8 : c.2332C>G, p.Arg778Gly**
**Exon 18 : c.3799G>A p.Asp1267Asn**
**intron 4: c.1708G>A, p.?**
**Exon 21 : c.4144G>T, p.Glu1382***
**Exon 21 : c.4144G>T, p.Glu1382***
**Exon 21 : c.4144G>T, p.Glu1382***
**Exon 14 : c.3207C>A, p.His1069Gln**
**Exon 16 : c.3532A>G, p.Thr1178Ala**
**Exon 18 : c.3799G>A p.Asp1267Asn**
**Exon 18 : c.3809A>G, p.Asn1270Ser**
**Exon 2 : c.331C>T, p.Gln111***
**Exon 20 : c.4092_4093del, p.Ser1365Cysfs*12**

**Intron 21 : c.4125-1G>T, p.?**
**Exon 18 : c.3809A>G, p.Asn1270Ser**
**Exon 14 : c.3207C>A, p.His1069Gln**
**Exon 19 : c.3965G>C p.Arg1322Pro**
**Exon 19 : c.3965G>C p.Arg1322Pro**
**Intron 4 : c.1707+2dupT, p.?**
**Intron 4 : c.1707+2dupT, p.?**
**Intron 4 : c.1707+2dupT, p.?**
**Exon 14 : c.3207C>A, p.His1069Gln**
**Exon 10 : c.2501T>A, p.Val834Asp**
**Exon 20 : c.4092_4093delGT, p.Ser1365Lysfs*12**
**Exon 20 : c.4092_4093del, p.Ser1365Cysfs*12**
**Exon 6 : c.3193G>C, p.Ala1065Pro**
**Intron 12 : c.2731-2A>G, p.Ser911Argfs*15**
**Intron 12 : c.2731-2A>G, p.Ser911Argfs*15**
**Exon 13 : c.3007G>A, p.Ala1003Thr**
**Exon 10 : c.2930C>T, p.Thr977Met**
**Exon 3 : c.1512dupT, p.Asn505***
**Exon 20 : c.4092_4093del, p.Ser1365Cysfs*12**
**Exon 13 : c.3057_3059del, p.His1019_Lys1020delinsGln**
**Exon 8 : c.2267C>T, p.Ala756Val**
**Exon 21 : c.4261C>T, p.Gln1421***
**Exon 16 : c.3443T>C, p.Ile1148Thr**
**Exon 18 : c.3818C>T, p.Pro1273Leu**
**Exon 13 : c.2930C>T, p.Thr977Met**

**Exon 21 : c.4261C>T, p.Gln1421***
**Exon 17 : c.3694A>C, p.Thr1232Pro**
**Exon 2 : c.676C>T,p.Arg226Trp and Exon 8 : c.2297C>G, p.Thr766Arg**
**Exon 2 : c.676C>T,p.Arg226Trp and Exon 8 : c.2297C>G, p.Thr766Arg**
**Exon 2 : c.676C>T,p.Arg226Trp and Exon 8 : c.2297C>G, p.Thr766Arg**
**Exon 2 : c.676C>T,p.Arg226Trp and Exon 8 : c.2297C>G, p.Thr766Arg**
**Exon 14 : c.865C>T, p.Gln289***
**Exon 19 : c.3974delT, p.Leu1325Argfs*5**
**Exon 19 : c.3974delT, p.Leu1325Argfs*5**
**Exon 19 : c.3974del p.Leu1325ArgfsTer5**
**Exon 19 : c.3974del p.Leu1325ArgfsTer5**
**Exon 14 : c.3207C>A, p.His1069Gln**
**Intron 7 : c.1946+5G>A, p.?**
**Exon 14 : c.3207C>A, p.His1069Gln**
**Exon 20 : c.4092_4093delGT, p.Ser1365Lysfs*12**
**Exon 20 : c.4092_4093del, p.Ser1365Cysfs*12**
**Exon 16 : c.3445G>C, p.Gly1149Arg**
**Exon 14 : c.3207C>A, p.His1069Gln**
**Exon 14 : c.3207C>A, p.His1069Gln**
**Intron 20 : c.4124+6T, p.?**
**Exon 14 : c.3207C>A, p.His1069Gln**
**Exon 14 : c.3207C>A, p.His1069Gln**
**Exon 19 : c.3955C>T, p.Arg1319***
**Intron 16 : c.3556+3A>G, p.?**
**Exon 16 : c.3547G>A, p.Ala1183Thr**

**Exon 8 : c.2128G>A, p.Gly710Ser**
**Exon 8 : c.2304dupC, p.Met769Hisfs*26**
**Exon 8 : c.2336G>A, p.Trp779***
**Exon 10 : c.2507G>A, p.Gly836Glu**
**Exon 14 : c.3207C>A, p.His1069Gln**
**Exon 2 : c.676C>T,p.Arg226Trp and Exon 8 : c.2297C>G, p.Thr766Arg**
**Exon 10 : c.2507G>A, p.Gly836Glu**
**Exon 10 : c.2507G>A, p.Gly836Glu**
**Exon 10 : c.2507G>A, p.Gly836Glu**
**Exon 10 : c.2507G>A, p.Gly836Glu**
**Exon 14 : c.3207C>A, p.His1069Gln**
**Exon 14 : c.3207C>A, p.His1069Gln**
**Exon 8 : c.2207C>T, p.Ala736Val**
**Exon 18 : c.3842G>A, p.Gly1281Asp**
**Exon 18 : c.3842G>A, p.Gly1281Asp**
**Exon 11 : c.2605G>A, p.Gly869Arg**
**Intron 15 : c.3412+2T>C, p.?**
**Intron 15 : c.3412+2T>C, p.?**
**Intron 15 : c.3412+2T>C, p.?**
**Exon 14 : c.3190G>A, p.Glu1064Lys**
**Exon 14 : c.3188C>T, p.Ala1063Val**
**Exon 16 : c.3422C>G, p.Pro1141Arg**
**Exon 18 : c.3842G>A, p.Gly1281Asp**
**Exon 8 : c.2332C>G, p.Arg778Gly**
**Exon 8 : c.2332C>G, p.Arg778Gly**

**Exon 4 : c.1651T>G, p.Phe551Val**
**Exon 13 : c.3059A>G, p.Lys1020Arg**
**Exon 13 : c.3059A>G, p.Lys1020Arg**
**Intron 1 : c.51+4A>T, p.?**
**Exon 18 : c.3842G>A, p.Gly1281Asp**
**Exon 2 : c.576_577insG, p.Gln193AlafsTer11**
**Exon 2 : c.1155_1157delinsC, p.Glu385AspfsTer19**
**Exon 4 : c.(1543+1_1544-1)_(1707+1_1708-1)del**
**Exon 4 : c.(1543+1_1544-1)_(1707+1_1708-1)del**
**Intron 1 : c.51+4A>T, p.?**
**Exon 13 : c.2930C>T, p.Thr977Met**
**Exon 8 : c.2332C>G, p.Arg778Gly**
**Exon 5 : c.1847G>A, p.Arg616Gln**
**Exon 13 : c.3008C>T, p.Ala1003Val**
**Exon 2 : c.314C>A, p.Ser105***
**Exon 2 : c.314C>A, p.Ser105***
**Intron 3 : c.1543+1G>T, p.?**
**Intron 5 : c.1869+2T>C, p.?**
**Intron 5 : c.1869+2T>C, p.?**
**Exon 18 : c.3809A>G, p.Asn1270Ser**
**Exon 14 : c.3207C>A, p.His1069Gln**
**Exon 14 : c.3207C>A, p.His1069Gln**
**Exon 19 : c.3955C>T, p.Arg1319***
**Exon 19 : c.3955C>T, p.Arg1319***
**Exon 10 : c.2501T>A, p.Val834Asp**

**Exon 14 : c.3207C>A, p.His1069Gln**
**Exon 14 : c.3082_3085delinsG, p.Lys1028SerfsX40**
**Exon 14 : c.3082_3085delinsG, p.Lys1028SerfsX40**
**Exon 7 : c.2007_2013del, p.Tyr670***
**Exon 14 : c.3207C>A, p.His1069Gln**
**Exon 14 : c.3207C>A, p.His1069Gln**
**Exon 14 : c.3207C>A, p.His1069Gln**
**Exon 14 : c.3207C>A, p.His1069Gln**
**Exon 14 : c.3207C>A, p.His1069Gln**
**Exon 14 : c.3207C>A, p.His1069Gln**
**Exon 14 : c.3207C>A, p.His1069Gln**
**Exon 13 : c.3007G>A, p.Ala1003Thr**
**Exon 8 : c.2128G>A, p.Gly710Ser**
**Intron 1 : c.51+4A>T, p.?**
**Exon 13 : c.3007G<A, p.Ala1003Thr and Exon14: c.3106G>A, p.Val1036Ile**
**Exon 13 : c.3007G<A, p.Ala1003Thr and Exon14: c.3106G>A, p.Val1036Ile**
**Exon 14 : c.3191A>C, p.Glu1064Ala**
**Exon 14 : c.3207C>A, p.His1069Gln**
**Exon 14 : c.3207C>A, p.His1069Gln**
**Exon 14 : c.3207C>A, p.His1069Gln**
**Exon 11 : c.2716G>C, p.Ala906Pro**
**Exon 17 : c.3659C>T, p.Thr1220Met**
**Exon 14 : c.3207C>A, p.His1069Gln**
**Exon 14 : c.3207C>A, p.His1069Gln**
**Exon 11 : c.2621C>T, p.Ala874Val**

**Exon 11 : c.2621C>T, p.Ala874Val**
**Exon 17 : c.3672_3674delinsTTCC, p.Lys1225Serfs*34**
**Exon 17 : c.3672_3674delinsTTCC, p.Lys1225Serfs*34**
**Exon 8 : c.2336G>A, p.Trp779***
**Exon 14 : c.3082_3085delinsG, p.Lys1028SerfsX40**
**Exon 21 : c.4144G>T; p.Glu1382***
**Exon 21 : c.4144G>T; p.Glu1382***
**Exon 18 : c.3809A>G, p.Asn1270Ser**
**Intron 5 : c.1869+5_1869+8delGTAA, p.?**
**Exon 8 : c.2333G>T, p.Arg778Leu**
**Exon 13 : c.2936T>A, p.Leu979Gln**
**Exon 14 : c.3104G>T, p.Gly1035Val**
**Exon 14 : c.3121C>T, p.Arg1041Trp**
**Exon 14 : c.3121C>T, p.Arg1041Trp**
**Intron 5 : c.1869+5_1869+8delGTAA, p.?**
**Intron 5 : c.1869+5_1869+8delGTAA, p.?**
**Exon 5 : c.1772G>A p.Gly591Asp**
**Exon 8 : c.2128G>A, p.Gly710Ser**
**Exon 16 : c.3461T>C, p.Leu1154Pro**
**Exon 16 : c.3461T>C, p.Leu1154Pro**
**Exon 16 : c.3461T>C, p.Leu1154Pro**
**Exon 18 : c.3842G>A, p.Gly1281Asp**
**Exon 17 : c.3617_3618insA, p.His1207Alafs*52**
**Exon 13 : c.3053C>T, p.Ala1018Val**
**Exon 8 : c.2128G>A, p.Gly710Ser**

**Exon 14 : c.3207C>A, p.His1069Gln**
**Exon 21 : c.4144G>T, p.Glu1382***
**Exon 20 : c.4092_4093delGT, p.Ser1365Lysfs*12**
**Exon 14 : c.3188C>T, p.Ala1063Val**
**Exon 14 : c.3188C>T, p.Ala1063Val**
**Exon 8 : c.2336G>A, p.Trp779***
**Exon 11 : c.2621C>T, p.Ala874Val**
**Exon 13 : c.3059A>G, p.Lys1020Arg**
**Exon 13 : c.3059A>G, p.Lys1020Arg**
**Exon 9 : c.2391_2393del, p.Leu798del**
**Exon 14 : c.3207C>A, p.His1069Gln**
**Exon 9 : c.2383C>T, p.Leu795Phe**
**Exon 9 : c.2383C>T, p.Leu795Phe**
**Exon 13 : c.3059A>G, p.Lys1020Arg**
**Intron 1 : c.51+5G>A, p.?**
**Exon 8 : c.2303C>T, p.Pro768Leu**
**Exon 13 : c.2930C>T, p.Thr977Met**
**Exon 8 : c.2304dupC, p.Met769Hisfs*26**
**Exon 14 : c.3207C>A, p.His1069Gln**
**Exon 17 : c.3694A>C, p.Thr1232Pro**
**Exon 17 : c.3694A>C, p.Thr1232Pro**
**Exon 17 : c.3694A>C, p.Thr1232Pro**
**Exon 10 : c.2519C>T, p.Pro840Leu**
**Exon 10 : c.2549C>T, p.Thr850Ile**
**Exon 10 : c.2549C>T, p.Thr850Ile**

**Exon 10 : c.2549C>T, p.Thr850Ile**
**Exon 2 : c.525dup, p.Val176SerfsX28**
**Exon 5 : c.1846C>T, p.Arg616Trp**
**Exon 5 : c.1846C>T, p.Arg616Trp**
**Exon 5 : c.1846C>T, p.Arg616Trp**
**Exons 2 - 5 : c.(51+1_52-1)_(1285+1_1286-1)del**
**Exons 2 - 5 : c.(51+1_52-1)_(1285+1_1286-1)del**
**Exon 6 : c.1934T>G, p.Met645Arg**
**Exon 2 : c.505C>T, p.Gln169Ter**
**Exon 10 : c.2532del, p.Val845Serfs*28**
**Exon 10 : c.2532del, p.Val845Serfs*28**
**Exon 10 : c.2532del, p.Val845Serfs*28**
**Intron 18 : c.3904-2A>G, p.?**
**Intron 18 : c.3904-2A>G, p.?**
**Exon 8 : c.2292C>T, p.Phe764Phe**
**Exon 14 : c.3207C>A, p.His1069Gln**
**Exon 8 : c.2332C>G, p.Arg778Gly**
**Exon 14 : c.3207C>A, p.His1069Gln**
**Exon 14 : c.3207C>A, p.His1069Gln**
**Exon 14 : c.3082_3085delinsG, p.Lys1028SerfsX40**
**Exon 5 : c.1745_1746delTA, p.Ile582Arg*25**
**Exon 4 : c.1705_1707+10del113, p.Thr569del**
**Exon 18 : c.3845dupT, p.Ala1283GlyfsX21**
**Exon 2 : c.1155_1157delinsC, p.Glu385AspfsX19**
**Exon 14 : c.3207C>A, p.His1069Gln**

**Exon 6 : c.1877G>C, p.Gly626Ala**
**Exon 13 : c.3008C>T, p.Ala1003Val**
**Exon 14 : c.3121C>T, p.Arg1041Trp**
**Exon 13 : c.3008C>T, p.Ala1003Val**
**Exon 8 : c.2333G>T, p.Arg778Leu**
**Exon 8 : c.2333G>T, p.Arg778Leu**
**Exon 8 : c.2333G>T, p.Arg778Leu**
**Exon 14 : c.3207C>A, p.His1069Gln**
**Exon 2 : c.676C>T,p.Arg226Trp and Exon 8 : c.2297C>G, p.Thr766Arg**
**Intron 7 : c.2122-8T>G, p.?**
**Exon 8 : c.2304dupC, p.Met769Hisfs*26**
**Intron 5 : c.1869+2T>C, p.?**
**Exon 16 : c.3532A>G, p.Thr1178Ala**
**Exon 14 : c.3207C>A, p.His1069Gln**
**Exon 14 : c.3207C>A, p.His1069Gln**
**Exon 14 : c.3207C>A, p.His1069Gln**
**Exon 7 : c.2046G>C, p.Met682Ile**
**Exon 2 : c.915T>A, p.Cys305***
**Exon 8 : c.2305A>G, p.Met769Val**
**Exon 8 : c.2304dupC, p.Met769Hisfs*26**
**Exon 18 : c.3818C>T, p,Pro1273Leu**
**Exon 14 : c.3207C>A, p.His1069Gln**
**Exon 8 : c.2304dupC, p.Met769Hisfs*26**
**Exon 8 : c.2304dupC, p.Met769Hisfs*26**
**Exon 3 : c.1369C>T, p.Gln457***

**Exon 3 : c.1369C>T, p.Gln457***
**Intron 2 : c.1286-416A>G, p.?**
**Intron 1 : c.51+4A>T, p.?**
**Exon 8 : c.2304C>T, p.Met 775Val**
**Exon 14 : c.3207C>A, p.His1069Gln**
**Exon 2 : c.1155_1157delinsC, p.Glu385Aspfs*19**
**Exon 4 : c.1625T>C, p.Ile542Thr**
**Intron 11 : c.2731-1G>A, p.?**
**Intron 11 : c.2731-1G>A, p.?**
**Exon 12 : c.2753A>G, p.Asp918Gly**
**Exon 16 : c.3556G>A, p.Gly1186Ser**
**Exon 10 : c.2519C>T, p.Pro840Leu**
**Exon 10 : c.2519C>T, p.Pro840Leu**
**Intron 1 : c.51+4A>T, p.?**
**Exon 3: c.1369C>T, p.Glu457***
**Intron 1 : c.51+4A>T, p.?**
**Exon 13 : c.3008C>T, p.Ala1003Val**
**Exon 2 : c.865C>T, p.Gln289***
**Intron 1 : c.51+4A>T, p.?**
**Exon 8 : c.2304dupC, p.Met769Hisfs*26**
**Exon 10 : c.2519C>T, p.Pro840Leu**
**Exon 14 : c.3207C>A, p.His1069Gln**
**Exon 14 : c.3207C>A, p.His1069Gln**
**Exon 14 : c.3207C>A, p.His1069Gln**
**Exon 16 : c.3551T>C, p.Ile1184Thr**

**Exon 16 : c.3551T>C, p.Ile1184Thr**
**Exon 7 : c.2007_2013del, p.Tyr670***
**Exon 8 : c.2305A>G, p.Met769Val**
**Exon 7 : c.2009_2015del, p.Tyr670***
**Exon 7 : c.2009_2015del, p.Tyr670***
**Exon 13 : c.2912C>T, p.Ala971Val**
**Exon 16 : c.3548C>G, p.Ala1183Gly**
**Exon 2 : c.331C>T, p.Gln111***
**Exon 14 : c.3207C>A, p.His1069Gln**
**Exon 8 : c.2305A>G, p.Met769Val**
**Intron 18 : c.3904-2A>G, p.?**
**Exon 14 : c.3207C>A, p.His1069Gln**
**Exon 14 : c.3207C>A, p.His1069Gln**
**Exon 14 : c.3207C>A, p.His1069Gln**
**Intron 1 : c.51+4A>T, p.?**
**Exon 14 : c.3207C>A, p.His1069Gln**
**Exon 8 : c.2304dupC, p.Met769Hisfs*26**
**Exon 14 : c.3207C>A, p.His1069Gln**
**Exon 14 : c.3207C>A, p.His1069Gln**
**Exon 14 : c.3207C>A, p.His1069Gln**
**Exon 18 : c.3818C>A, p.Pro1273Gln**
**Exon 18 : c.3842G>A, p.Gly1281Asp**
**Exon 6 : c.1877G>C, p.Gly626Ala**
**Exon 13 : c.3007G>C, p.Ala1003Pro**
**Exon 14 : c.3207C>A, p.His1069Gln**

**Exon 14 : c.3207C>A, p.His1069Gln**
**Exon 14 : c.3207C>A, p.His1069Gln**
**Exon 14 : c.3207C>A, p.His1069Gln**
**Exon 14 : c.3207C>A, p.His1069Gln**
**Exon 14 : c.3207C>A, p.His1069Gln**
**Exon 14 : c.3207C>A, p.His1069Gln**
**Exon 14 : c.3207C>A, p.His1069Gln**
**Exon 14 : c.3207C>A, p.His1069Gln**
**Exon 14 : c.3207C>A, p.His1069Gln**
**Exon 14 : c.3207C>A, p.His1069Gln**

		Number of values	Mean	Median	Minimum	Maximum	2.5% Percentile	97.5% Percentile	p-value
**Total serum copper (µmol/L)**	F	126	17.5	16.6	7.6	35.4	10.5	31.7	0.0098
	M	89	15.8	14.9	2.9	28.4	7.8	26.3	
**Exchangeable Copper (CuEXC) (µmol/L)**	F	126	0.88	0.86	0.45	1.90	0.48	1.45	0.4099
	M	89	0.84	0.84	0.49	1.38	0.52	1.29	
**Relative Exchangeable copper (REC) (%)**	F	126	5.23	5.00	2.20	12.80	2.62	9.77	0.0537
	M	89	5.81	5.40	2.40	19.10	2.50	9.30	
**Ceruloplasmin (g/L)**	F	117	0.276	0.270	0.170	0.479	0.190	0.410	0.0083
	M	80	0.251	0.239	0.090	0.390	0.142	0.390	
**Urinary Copper (µmol/24h)**	F	51	0.19	0.15	0.02	0.97	0.02	0.87	0.013
	M	41	0.33	0.19	0.08	2.07	0.08	2.07	
**Age (years)**	F	126	28	27	2	77	3	64	0.0719
	M	89	24	21	2	72	3	66	
**AST (IU/L)**	F	99	25	22	15	52	16	41	< 0.0001
	M	75	33	29	14	200	16	118	
**ALT (IU/L)**	F	99	19	18	6	69	9	35	0.0114
	M	75	30	20	6	256	8	256	

Table S2. Results of copper metabolism assessment in controls, with comparisons by sex

Serum copper and CuEXC conversion: μmol/L × 6.3546 = μg/dL. F: female; M: male;

		Number of values	Mean	Median	Minimum	Maximum	2.5% Percentile	97.5% Percentile	p-value
**Age (years)**	<16 y	72	7	7	2	15	2	15	
	≥16 y	143	36	35	16	77	16	69	
**Total serum copper (µmol/L)**	<16 y	72	17	17	8	28	9	27	
	≥16 y	143	17	16	3	35	8	30	0.1338
**Exchangeable Copper (CuEXC) (µmol/L)**	<16 y	72	0.79	0.77	0.48	1.32	0.50	1.21	
	≥16 y	143	0.90	0.88	0.45	1.90	0.53	1.41	0.0006
**Relative Exchangeable copper (REC) (%)**	<16 y	72	4.91	4.70	2.40	12.80	2.48	10.74	
	≥16 y	143	5.75	5.40	2.20	19.10	3.26	9.42	0.0004
**Ceruloplasmin (g/L)**	<16 y	65	0.278	0.280	0.170	0.400	0.195	0.394	
	≥16 y	132	0.259	0.250	0.090	0.479	0.145	0.406	0.0243
**Urinary Copper (µmol/24h)**	<16 y	35	0.24	0.14	0.05	2.07	0.05	2.07	0.1344
	≥16 y	57	0.26	0.18	0.02	2.02	0.02	1.46	
**AST (IU/L)**	<16 y	60	32	33	16	55	17	53	< 0.0001
	≥16 y	114	26	22	14	200	16	109	
**ALT**	<16 y	60	17	18	6	31	6	28	0.0035

**(IU/L)**	≥16 y	114	27	20	9	256	11	163	

Table S3. Results of copper metabolism assessment in controls, with comparisons across age groups.

Serum copper and CuEXC conversion: µmol/L × 6.3546 = µg/dL. F: female; M: male.

Group	Gender	Age	Variant 1	Variant 2	Phenotype	Total Cu[1] (µmol/L)	Total Cu[1] (µg/dL)	CuEXC[2] (µmol/L)	CuEXC[2] (µg/dL)	REC[3] (%)	Cp[4] (g/L)	24 hr-UCE[5] (µmol/24h)	AST (U/L)	ALT (U/L)	PT (%)	Platelet count (G/L)	Hemoglobin	KFR	
WD	F	30	c.3007G>A	c.3106G>A	Hepatic	10.61	67.4	1.37	8.7	12.91	0.16	NA	52	55	71	76	12.6	Yes	False negative
WD	F	1	c.1708-1G>A	c.3620A>G. p.His1207Arg	Asymptomatic	20.55	130.6	0.75	4.8	3.65	0.47	NA	42	33	100	233	11.3	NA	False negative
WD	M	13	c.1708-1G>A	c.1708-1G>A	Hepatic	11.50	73.1	1.05	6.7	9.13	0.18	NA	43	88	91	221	15.8	No	False negative
WD	F	8	c.2128G>A. p.Gly710Ser	c.3505A>G. p.Met1169Val	Hepatic	13.66	86.8	1.3	8.3	9.52	0.22	1.57	56	80	88	290	14.1	No	False negative
WD	M	53	c.2804C>T p.Thr935Met	c.2804C>T p.Thr935Met	Asymptomatic	10.00	63.5	1.15	7.3	11.50	0.22	0.39	28	31	93	267	14.5	No	False negative
WD	F	35	c.2804C>T p.Thr935Met	c.2804C>T p.Thr935Met	Asymptomatic	14.00	89	0.97	6.2	6.93	0.28	0.33	20	18	87	257	13.9	No	False negative
WD	F	12	c.1708-1G>A	c.1708-1G>A	Hepatic	12.47	79.2	1.4	8.9	11.23	0.18	2.88	196	336	88	328	11.8	No	False negative
WD	F	21	c.670dup p.Ile224Asnfs*2	c.2046G>C. p.Met682Ile	Asymptomatic	15.20	96.6	0.89	5.7	5.86	0.22	0.32	21	16	100	280	13.9	No	False negative
WD	M	8	c.2128G>A p.gLY710Ser	c.2305A>G. p.Met769Val	Hepatic	15.79	100.3	2.10	13.3	13.30	0.25	2.7	105	253	91	346	13.2	No	False negative
Control	M	21	NA	NA	CDG	2.88	18.3	0.55	3.5	19.1	0.09	0.5	200	150	100	NA	NA	NA	False positive

																		(CDG diagnosis)

Table S4. Main clinical, biological and genetic features associated with false negative and false positive REC results.

F: Female; M: Male; Total Cu: Total serum copper; CuEXC: Exchangeable copper; REC: Relative exchangeable copper; Cp: Ceruloplasmin; 24 hr-UCE: 24-hour urinary copper excretion; AST: Aspartate aminotransferase; ALT: Alanine aminotransferase; PT: Prothrombin rate; KFR: Kayser Fleischer Ring; CDG: Congenital Disorder of Glycosylation.

Reference ranges were determined across all control samples

[1] Reference range: 8.5-28.2 µmol/L (24-179.2 µg/dL)

[2] Reference range: 0.50-1.38 µmol/L (3.2-8.8 µg/dL)

[3] Reference range: 2.6-9.5%

[4] Reference range: 0.17-0.4 g/L

[5] Reference range: 0.03-1.679 µmol/24h

Table S5. Comparison of total copper, exchangeable copper (CuEXC), and relative exchangeable copper (REC) levels in controls and heterozygous carriers (HTZ) across two time periods (2009–2014 vs. after 2015).

		Period	n	Median	Mean	Std. Deviation	P value
Total copper (µmol/L)	Controls	2009-2014	59	16,2	16,24	3,356	0,895
		After 2015	102	15,75	16,52	4,309	
	HTZ	2009-2014	147	14,9	15,52	4,277	0,0983
		After 2015	123	14,01	14,74	4,128	
Exchangeable copper (µmol/L)	Controls	2009-2014	59	0,91	0,92	0,18	0,0218
		After 2015	102	0,83	0,85	0,21	
	HTZ	2009-2014	147	0,88	0,90	0,20	0,5336
		After 2015	121	0,89	0,94	0,29	
Relative exchangeable copper (%)	Controls	2009-2014	59	5,5	5,84	1,52	0,0889
		After 2015	102	5,1	5,46	1,93	
	HTZ	2009-2014	147	5,9	6,10	1,78	0,0508
		After 2015	123	6,3	6,68	2,52	
