# Supplementary material for: Relative exchangeable copper: A highly specific and sensitive biomarker for Wilson disease diagnosis
Source: JHEP Rep. 2025 Jul 31;7(10):101537. doi: 10.1016/j.jhepr.2025.101537 (PMC12446559; doi:10.1016/j.jhepr.2025.101537)
Supplement: Multimedia component 4 [file mmc4.pdf]

Research article

JHEP Reports

Relative exchangeable copper: A highly specific and sensitive biomarker for Wilson disease diagnosis

Authors

Nouzha Djebrani-Oussedik, Clément Desjardins, Mickaël Alexandre Obadia, …, Joël Poupon, Dominique Debray, Aurélia Poujois

Correspondence

apoujois@for.paris (A. Poujois).

Graphical abstract

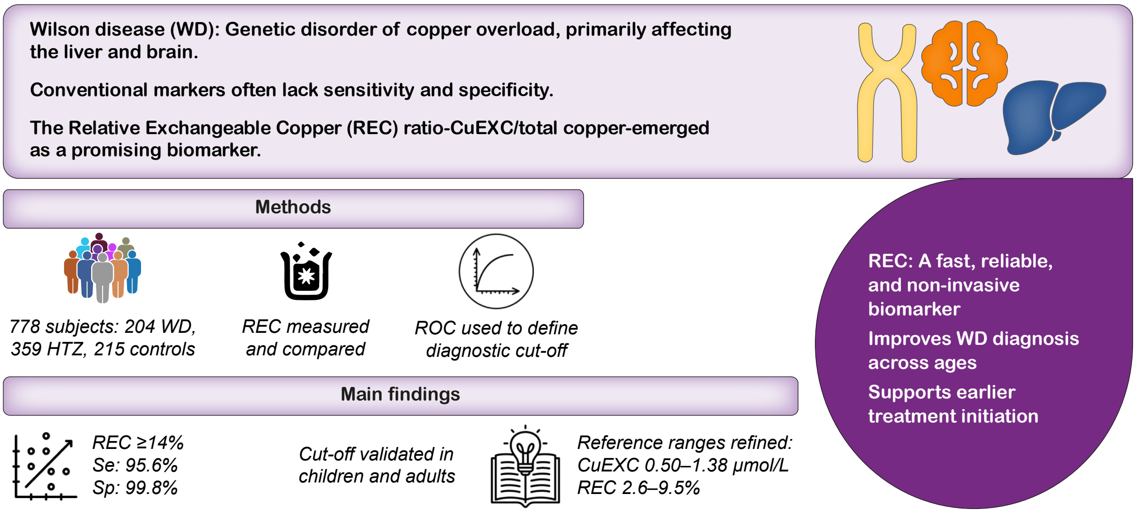

Highlights:

- REC is a robust biomarker for Wilson disease with high diagnostic accuracy.
- A 14% REC cut-off yields 95.6% sensitivity and 99.8% specificity across ages.
- REC helps avoid invasive liver biopsy in patients with unclear copper status.
- REC enables earlier diagnosis and treatment, improving patient outcomes.

Impact and implications:

The study established relative exchangeable copper (REC) as a robust diagnostic biomarker for Wilson disease (WD), demonstrating high sensitivity and specificity across age groups in a large cohort including 204 patients with WD. By refining the optimal REC cut-off, this research provides crucial insights for improving WD diagnostic accuracy and patient outcomes. These findings confirm the need to incorporate REC into routine clinical practice and WD management guidelines, potentially reducing the reliance on invasive liver biopsies to assess hepatic copper levels. Consequently, this advancement in diagnostic methodology could facilitate earlier detection and treatment, thereby preventing irreversible tissue damage and enhancing the quality of life for patients with WD.

https://doi.org/10.1016/j.jhepr.2025.101537
© 2025 The Authors. Published by Elsevier B.V. on behalf of European Association for the Study of the Liver (EASL). This is an open access article under the CC BY license (http://creativecommons.org/licenses/by/4.0/).

JHEP|Reports

Relative exchangeable copper: A highly specific and sensitive biomarker for Wilson disease diagnosis

Nouzha Djebrani-Oussedik[1,2,†], **Clément Desjardins**[1,3,†], Mickaël Alexandre Obadia[1,3], Djamila Rahli[1], Corinne Collet[4], France Woimant[1], Joël Poupon[1,2], Dominique Debray[1,3], Aurélia Poujois[1,3,*]

JHEP Reports **2025**. vol. 7 | 1–9

Check for updates

Background & Aims: Wilson disease (WD) is an autosomal recessive disorder characterized by copper accumulation in various organs, primarily the liver and brain. Standard assessment of copper metabolism includes total serum copper, serum ceruloplasmin, and urinary copper excretion. Quantitative measurement of non-ceruloplasmin-bound copper, known as exchangeable copper (CuEXC), was developed in 2009. Subsequently in 2011, relative exchangeable copper (REC), defined as the ratio of CuEXC to total serum copper, was proposed as a diagnostic biomarker. This study aimed to validate the REC cut-off for the diagnosis of WD in a large cohort and to refine the reference ranges for CuEXC.

Methods: Data were collected from 778 individuals at the French National Reference Centre for WD from January 2009 to 2025. The cohort included 204 patients with WD, 359 healthy heterozygous carriers, and 215 controls. All participants underwent clinical evaluation, assessment of copper metabolism, including CuEXC and REC, and genetic testing for *ATP7B*. Receiver operating characteristic curve analysis was used to assess the diagnostic performance of REC and to determine the optimal cut-off for diagnosing WD.

Results: Patients with WD had significantly higher CuEXC and REC values compared with heterozygous carriers and controls. The optimal REC cut-off for diagnosing WD was identified as 14% with 95.6% sensitivity and 99.8% specificity. This cut-off was validated in both pediatric and adult subgroups with similar sensitivity and specificity. Reference ranges for CuEXC (0.50–1.38 μmol/L) and for REC (2.6–9.5%) were refined using control group data. Age-specific ranges were also determined.

Conclusion: This study supports the use of REC in clinical practice and confirms its central role in the diagnostic algorithm for WD, as recognized in the recently published EASL 2025 guidelines.

© 2025 The Authors. Published by Elsevier B.V. on behalf of European Association for the Study of the Liver (EASL). This is an open access article under the CC BY license (http://creativecommons.org/licenses/by/4.0/).

Introduction

Wilson disease (WD) is an autosomal recessive disorder of copper (Cu) metabolism leading to Cu accumulation in various organs, primarily the liver and brain.[1–4] Failure to diagnose WD can result in lost opportunities for initiating treatment, potentially leading to irreversible tissue damage and even death.[5,6] A Leipzig score ≥4 supports the diagnosis of WD and is based on a combination of clinical findings, including: neurological symptoms and/or presence of Kayser–Fleischer rings (KFR); abnormal brain magnetic resonance imaging (MRI); elevated hepatic Cu content; laboratory abnormalities, such as hemolytic anemia, low serum ceruloplasmin (Cp), increased urinary Cu excretion (UCE), and the identification of two biallelic pathogenic *ATP7B* variants.[7,8] Calculated serum non-Cp-bound copper (NCC), an estimate of the toxic unbound Cu fraction, has traditionally been used as a diagnostic marker.[9–12] However, calculation of NCC is often unreliable, with up to 20% of results yielding negative values.[9–12] This inaccuracy stems from its dependence on both Cp and total serum Cu concentrations, the precision of which directly affects the calculated NCC.

A method for directly measuring NCC, without relying on Cp measurements, was developed and has been routinely used in France since 2011.[10,13] More recently, this approach has been implemented in other countries, including Spain.[14] This biomarker, known as 'exchangeable copper' (CuEXC) represents the labile fraction of Cu complexed with albumin and other peptides, which readily exchanges in the presence of high-affinity Cu-chelating agents.[15] Initial studies based on small cohorts showed that the ratio of CuEXC to total serum Cu, referred to as relative exchangeable copper (REC), is a valuable parameter in the diagnosis of WD. Hence, REC has been introduced as a specific diagnostic biomarker for WD[13,14,16,17] and is now incorporated into the diagnostic algorithm recommended by the 2025 EASL-ERN Clinical Practice Guidelines on Wilson's disease.[18]

Normal ranges of CuEXC in healthy individuals have previously been established as 0.62–1.15 μmol/L (3.94–7.31 μg/dl).[10] A REC value >18.5% was previously reported to provide

* Corresponding author. Address: Rothschild Foundation Hospital, 29 rue Manin, 7019 Paris, France. Tel.: +33-1-48-03-65-65; Fax: +33-1-48-03-65-27.
E-mail address: apoujois@for.paris (A. Poujois).
† Co-first authors.
https://doi.org/10.1016/j.jhepr.2025.101537

100% sensitivity (Se) and specificity (Sp) for the diagnosis of WD in a cohort of 16 patients with WD.[13] In addition, REC was shown to effectively distinguish WD-related liver involvement from other liver disorders in both pediatric and adult populations.[16] In the context of family screening, a lower REC cut-off of 15% was proposed based on a small cohort of five patients with WD.[17] The primary limitation of these studies was their small sample sizes.

In this study, we aimed to validate the REC cut-off for the diagnosis of WD in a large cohort of 204 patients with WD compared with healthy heterozygous (HTZ) carriers and control individuals, and to refine the normal reference ranges for CuEXC.

Materials and methods

Study population

Data were collected from the French National Reference Centre for Wilson Disease at Lariboisière Hospital and Rothschild Foundation Hospital, Paris. Between January 2009 and January 2025, a total of 778 individuals, including patients with suspected WD, and first- or second-degree relatives of index cases (siblings, parents, children, uncles or aunts, and cousins), underwent Cu metabolism assessment. All individuals had neurological and hepatic examinations, as well as biological investigations, including measurements of aminotransferase levels, prothrombin time, complete blood count, total serum Cu (total Cu) and CuEXC. Cp levels and 24-h UCE were also recorded when available. All data were collected at the time of diagnosis or initial screening before the initiation of any treatment. Genetic analyses were performed by bi-directional sequencing of the 21 exons and intron–exon boundary regions of *ATP7B* gene. For patients with WD with only one *ATP7B* variant or without variant detected by sequencing, a multiplex ligation-dependent probe amplification (MLPA) assay was performed using the SALSA MLPA P098 for Wilson Disease kit (MRC-Holland, Amsterdam, The Netherlands) to detect large deletions. Next-generation sequencing has been used since 2017 to detect *ATP7B* variants.[19] Most of the variants identified are known to be disease-causing mutations, as reported in either the WilsonGen database[20] or Human Gene Mutation Database (www.hgmd.cf.ac.uk). All variants were classified as pathogenic or likely pathogenic according to the American College of Medical Genetics and Genomics guidelines.[21] A diagnosis of WD was established with a Leipzig score ≥4 and was confirmed by experts from the French National Reference Centre for Wilson Disease. Based on molecular analysis, biological and clinical data, participants were categorized into three groups: (1) controls (no *ATP7B* variants); (2) healthy HTZ carriers; and (3) patients with WD.

All participants were fully informed and provided written consent for genetic testing. The study was approved by the Rothschild Foundation Hospital review board (IRB00012801; study no. CE_20240723_8_APS).

Determination of biological parameters

The method used for determining CuEXC was as previously described.[10] The ultrafiltration step, used to isolate the NCC fraction, was rigorously standardized. Serum samples were diluted 1:1 with EDTA (3 g/L) and incubated for exactly 1 h at room temperature before ultrafiltration using Amicon Ultra-4 units (Millipore, Molsheim, France). This protocol minimizes variability and has been validated through interlaboratory comparisons.

Cu quantification for both CuEXC and total Cu evolved over the course of the study in accordance with laboratory advances. Initially, electrothermal atomic absorption spectrometry was used; later, analyses were conducted using inductively coupled plasma-mass spectrometry (ICP-MS) with the Elan DRCe®, NexION 2000® and Nexion 5000® spectrometers (Perkin Elmer, Les Ulis, France). This methodological transition was accompanied by a rigorous validation process, including comparative analysis to ensure consistency in results and reference values. The REC was calculated as the ratio of CuEXC to total serum Cu levels. Urinary Cu concentrations were measured using ICP-optical emission spectrometry with the iCAP 6300DV spectrometer (ThermoFisher Scientific, Les Ulis, France). Serum Cp levels were determined with the immunonephelometric method (C4000 and Alinity Abbott, Rungis, France). NCC was calculated using Equation 1[22]:

$$NCC\ (\mu mol/L) = total\ Cu\ (\mu mol/L) - 0.049 \times Cp\ (mg/L) \quad (1)$$

Statistical analyses

Statistical analyses were performed using R version 4.4.0 and GraphPad Prism version 9.2.0. Continuous variables (CuEXC, total Cu, UCE, and Cp levels) were summarized by the number of patients, mean, median, maximum, minimum, and percentile. Because of the non-normal distribution of variables, intergroup comparisons were conducted using the Kruskal–Wallis ANOVA (Mann-Whitney *U* test).

Median Cu values in the control group were reported, along with the calculated reference interval (2.5–97.5 percentiles) stratified by sex, age group, and genetic status. Receiver operating characteristic (ROC) curve analysis was performed on the entire study population to determine the Se, Sp, and predictive values of the REC. The area under the ROC curve was calculated, and the optimal cut-off value for the diagnosis of WD was determined by maximizing the Youden index. CIs were fixed at 95%. The optimal cut-off was subsequently validated in two independent groups, pediatric (<16 years old) and adult (≥16 years old), to ensure the generalizability of the findings across age groups. All p values were based on two-tailed tests, and results with p <0.05 were considered statistically significant.

Results

Baseline population characteristics

In total, 778 individuals were included in the study, of whom 204 (26%) met the diagnostic criteria for WD as defined by a Leipzig score ≥4 (mean age 28 years, 105 women), including 91 children <16 years old (mean age 10 years) and 113 adults ≥16 years old (mean age 31 years). Of the total cohort (778), 359 individuals (46%) were healthy HTZ carriers (mean age 27 years, 182 women, 161 children) and 215 (28%) were controls who did not carry any *ATP7B* variant (mean age 26 years, 126 women, 72 children) (Table 1).

Table 1. Baseline characteristics of the three study groups.

Characteristic	Controls (n = 215)	HTZ carriers (n = 359)	WD (n = 204)
Sex, n (%)			
Female	126 (58)	183 (51)	104 (51)
Male	89 (42)	176 (49)	100 (49)
Age at diagnosis or assessment (years)	26.3 (10.5–39)	26.6 (6–45)	21 (12–28.3)
Mutations, n (%)			
0	215 (100)	0 (0)	1 (0.5)
1	0 (0)	350 (97)	8 (4)
2	0 (0)	9[‡] (3)	186 (91)
3 or 4	0 (0)	0 (0)	9[‡] (4.5)
KFR	—	—	72 (35)
NA	—	—	8
Phenotype			
Asymptomatic	—	—	31 (15)
Hepatic	—	—	105 (51)
with KFR	—	—	21 (20)
without KFR	—	—	84 (80)
Hepatoneuropsychiatric	—	—	64 (31)
with KFR	—	—	54 (81)
without KFR	—	—	13 (19)
NA	—	—	4 (2)
Leipzig score, n (%)			
0	206 (95.8)	0 (0)	0 (0)
1	6[*] (2.8)	314 (87.5)	0 (0)
2	3[†] (1.4)	43 (11.9)	0 (0)
3	0 (0)	2 (0.6)	0 (0)
≥4	0 (0)	0 (0)	204 (100)
Biological liver data, mean [IQR]			
AST (IU/L)	28.4 [20–32]	30.5 [24–35]	83.8 [32.7–121.5]
ALT (IU/L)	23.6 [23–15]	24.0 [15.8–26.3]	114.5 [31–144.5]
Prothrombin rate (%)	93.7 [89–100]	96.3 [90–100]	72.2 [57–91]
Platelet count (G/L)	280.3 [234–323.5]	294.1 [234.5–336]	198.8 [119–261]

Values are numbers (%) or mean (IQRs).
ALT, alanine aminotransferase; AST, aspartate aminotransferase; KFR, Kayser–Fleischer ring; NA, not available; WD, Wilson disease.
*Four had slightly decreased ceruloplasmin levels <0.2 g/L, and two others increased 24-h urinary copper excretion one to two times the upper limit of normal.
†One had decreased ceruloplasmin level <0.1 g/L and was found to have a congenital disorder of glycosylation; two others had 24-h urinary copper excretion more than twice the upper limit of normal.
‡Two variants on the same allele.

Among the 204 patients with WD, 15% (n = 31) were asymptomatic with normal transaminase levels. Hepatic involvement (*i.e.* hepatic form) was observed in 51% (n = 105), including 21 (20%) with KFR, and liver and brain involvement (*i.e.* hepatoneuropsychiatric form) was present in 31% (n = 64), of whom 13 (20%) had no KFR. Phenotypic characterization was unavailable for four patients (2%).

Results of Cu metabolism assessment (including total Cu, Cp, CuEXC, 24-h UCE and REC values) are presented in Fig. 1 and Table 2. Genetic data from patients with WD and HTZ carriers are shown in Table S1.

Copper metabolism assessment in the control group

The median total Cu level was 16.0 µmol/L (101.7 µg/dl), with a calculated reference interval (2.5–97.5th percentiles) ranging from 8.5 to 28.2 µmol/L (24–179.2 µg/dl). The median total Cu level was significantly higher in women compared with men (16.6 *vs.* 14.9 µmol/L, p <0.01). There was no significant difference in Cu levels between children (<16 years) and adults (≥16 years) (see also Table 2; Tables S2 and S3).

The median Cp level was 0.26 g/L, with a calculated reference interval of 0.17–0.4 g/L. The median Cp level was significantly higher in women compared with men (0.28 *vs.* 0.25 g/L; p <0.01) and in children compared with adults (0.28 *vs.* 0.26 g/L; p <0.05).

The median 24-h UCE was 0.17 µmol/24 h, with a calculated reference interval of 0.03–1.68 µmol/24 h. The median 24-h UCE was higher in men compared with women (0.19 *vs.* 0.15 µmol/24 h; p <0.05).

The median CuEXC level was 0.84 µmol/L (5.3 µg/dl), with a calculated reference interval of 0.50–1.38 µmol/L (3.2–8.8 µg/dl). The median CuEXC level was significantly higher in adults (0.88 µmol/L, n = 143) compared with children (0.77 µmol/L, n = 72; p <0.001). No significant sex-based difference was observed.

The median REC value was 5.1% with a calculated reference interval of 2.6–9.5%. The median REC value was higher in adults compared with children (5.4% *vs.* 4.7%; p <0.05). No significant sex-based difference was observed.

To address potential procedural variability, we analyzed CuEXC values across two time periods and observed a slight decrease in control group values after 2015 compared with the period before 2015 (Table S5). REC values remained stable over time.

Copper metabolism assessment according to genetic status

ATP7B mutation status significantly influenced all Cu metabolism parameters (Table 2). Compared with healthy HTZ carriers and controls, patients with WD had significantly lower

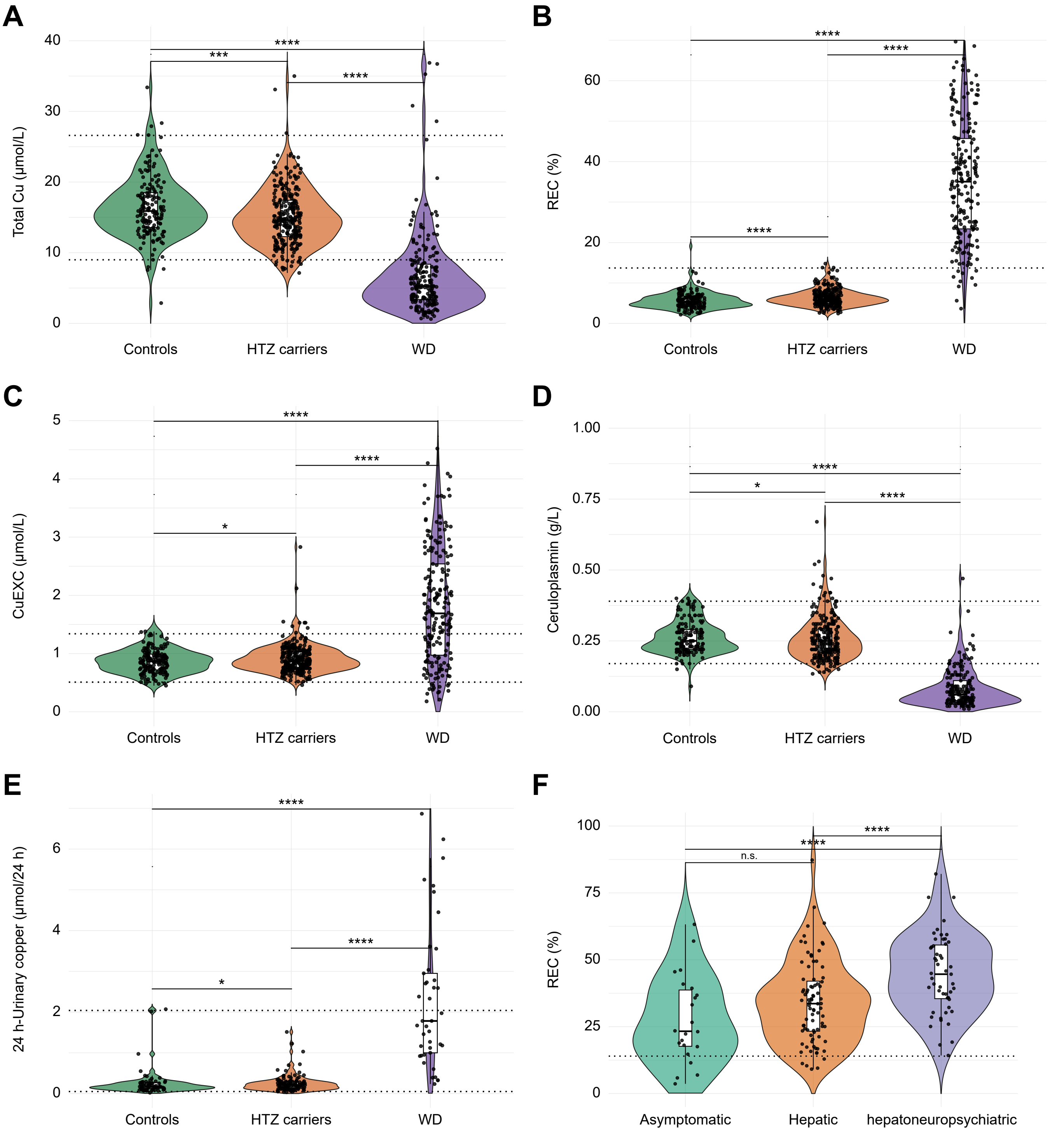

Fig. 1. Diagnostic tests for controls (green, n = 215), healthy HTZ carriers (orange, n = 359) and patients with WD (purple, n = 204)" (A) Total serum Cu, (B) REC, (C) CuEXC, (D) immunological Cp, (E) 24-h urinary Cu excretion, and (F) REC across WD phenotypes (asymptomatic, hepatic, and hepatoneuropsychiatric). Broken lines represent the cut-off obtained from receiver operating characteristic analysis for REC and reference ranges obtained for other parameters. Given the non-normal distribution of variables, intergroup comparisons were conducted using the Kruskal–Wallis ANOVA (Mann-Whitney *U* test); **p* <0.05; ****p* <0.001; *****p* <0.0001; ns: not significant. Cp, ceruloplasmin; Cu, copper; CuEXC, exchangeable copper levels; HTZ carriers, heterozygous carriers; REC, relative exchangeable copper; WD, Wilson disease.

Table 2. Results of Cu assessment in controls, healthy HTZ carriers, and patients with WD.

Group	No.	Mean	Median	Minimum	Maximum	2.5% percentile	97.5% percentile	*p* value (*vs.* controls)
Total serum Cu (µmol/L)								
Controls	215	16.8	16.0	2.9	35.4	8.5	28.2	—
HTZ carriers	359	15.4	14.9	7.1	35.0	8.7	24.5	0.0001
Patients with WD	204	7.1	5.3	0.6	42.2	1.1	30.6	<0.0001
Total serum Cu (µg/dl)								
Controls	215	106.8	101.7	18.4	224.9	54.0	179.2	—
HTZ carriers	359	97.9	94.7	45.2	222.4	55.3	155.7	0.0001
Patients with WD	204	45.2	33.7	3.8	268.2	7.0	194.5	<0.0001
CuEXC (µmol/L)								
Controls	215	0.86	0.84	0.45	1.90	0.50	1.38	—
HTZ carriers	359	0.91	0.87	0.16	4.37	0.56	1.47	0.0327
Patients with WD	204	2.49	1.80	0.18	23.49	0.34	14.28	<0.0001
CuEXC (µg/dl)								
Controls	215	5.5	5.3	2.9	12.1	3.2	8.8	—
HTZ carriers	359	5.8	5.8	1.0	27.8	3.6	9.3	0.0327
Patients with WD	204	15.8	11.4	1.1	149.3	2.2	90.7	<0.0001
Relative exchangeable copper (%)								
Controls	215	5.47	5.10	2.20	19.10	2.60	9.48	—
HTZ carriers	359	6.20	5.90	1.60	13.80	3.10	11.30	<0.0001
Patients with WD	204	36.15	35.25	3.65	87.27	9.73	69.53	<0.0001
Ceruloplasmin (g/L)								
Controls	197	0.266	0.260	0.090	0.479	0.169	0.400	—
HTZ carriers	329	0.258	0.250	0.134	0.670	0.160	0.435	0.0256
Patients with WD	185	0.081	0.060	0.010	0.470	0.017	0.244	<0.0001
24-h urinary Cu (µmol/24 h)								
Controls	92	0.25	0.17	0.02	2.07	0.03	1.68	—
HTZ carriers	153	0.32	0.21	0.03	4.64	0.05	1.66	0.0226
Patients with WD	47	2.62	1.78	0.23	11.40	0.25	10.81	<0.0001

Given the non-normal distribution of variables, intergroup comparisons were conducted using the Kruskal–Wallis ANOVA (Mann-Whitney *U* test). Serum Cu and CuEXC conversion: µmol/Lx6.3546 = µg/dl.
Cu, copper; CuEXC, exchangeable copper; HTZ, heterozygous; WD, Wilson disease.

median total Cu levels (5.3 *vs.* 14.9 µmol/L and 16 µmol/L; *p* <0.0001) and median Cp levels (0.06 g/L *vs.* 0.25 and 0.26 g/L; *p* <0.0001). Conversely, compared with healthy HTZ carriers and controls, patients with WD had significantly higher median CuEXC levels (1.8 µmol/L *vs.* 0.87 µmol/L; *p* <0.05, and 0.84 µmol/L; *p* <0.001) and median REC values (35.25% *vs.* 5.9% and 5.1%; *p* <0.0001, respectively). Among patients with WD, 4.9% (n = 10/204) had normal total Cu levels, 1.6% (n = 3/185) had elevated Cp levels, 3.8% (n = 7/185) had normal Cp levels, and 44.7% (n = 21/47) had normal 24-h UCE (<0.6 µmol/24 h).

Compared with controls, HTZ carriers had a lower median total Cu (14.9 µmol/L *vs.* 16.0 µmol/L; *p* <0.0001), higher median CuEXC level (0.87 µmol/L *vs.* 0.84 µmol/L; *p* <0.05), higher REC value (5.9% *vs.* 5.1%; *p* <0.05), and higher 24-h UCE (0.21 µmol/24 h *vs.* 0.17 µmol/24 h; *p* <0.05). CuEXC values were not significantly different after 2015 compared with the period before 2015 (Table S5).

Some HTZ carriers and controls exhibited abnormal total Cu and Cp levels. Among HTZ carriers, 20% (n = 71/359) had low total Cu (<12 µmol/L), whereas 5.6% (n = 20/359) had elevated total Cu (>22 µmol/L), 17.9% (n = 59/329) had low Cp levels (<0.20 g/L) and 50% (n = 77/153) had increased 24-h UCE (>0.6 µmol/24 h). Among controls, 8.8 % (n = 19/215) had low total Cu, whereas 11.6% (n = 25/215) had elevated total Cu, 6% (n = 12/197) had low Cp levels, and 33.7% (n = 31/92) had increased 24-h UCE.

A Cp cut-off <0.2 g/L for WD diagnosis yielded a Se of 95.2% (91.8–98.7%), Sp of 81.9% (78–85.8%), positive predictive value (PPV) of 67.6% (61.2–74%) and negative predictive value (NPV) of 82% (78–86.2%).

A 24-h UCE Cu cut-off >0.6 µmol/24 h yielded a Se of 88% (84–93%), Sp of 95% (92–98%), PPV of 83% (71–93%), and NPV of 96% (94–99%).

A CuEXC cut-off >1.15 µmol/L (7.3 µg/dl) yielded a Se of 74% (67.6–79.6%), Sp of 90.8% (88.1–92.9%), PPV of 74% (67.6–79.6%), and NPV of 90.8% (88.1–92.9%).

NCC calculation was uninterpretable, yielding negative results in 8% of controls (n = 16/197), 8% of HTZ carriers (n = 26/329) and 27% of patients with WD (n=50/185). The median NCC (when positive) was 3.97, 3.25, and 3.07 in controls, HTZ carriers, and patients with WD, respectively, and, therefore, similar across the three subgroups.

REC cut-off for the diagnosis of WD

REC values were analyzed across the entire study population (Fig.1). ROC curve analysis comparing patients with WD, HTZ carriers, and controls identified 13.93% as the optimal cut-off value for REC. At this threshold, the test demonstrated a Se of 95.6% (91.8–97%) and Sp of 99.8% (99.8–100%) (Fig. 2). The PPV was 99.1% (98.9–100%) and the NPV was 98.6% (97.6-99.5%).

REC values were also analyzed independently in pediatric and adult subgroups, and the results closely mirrored those

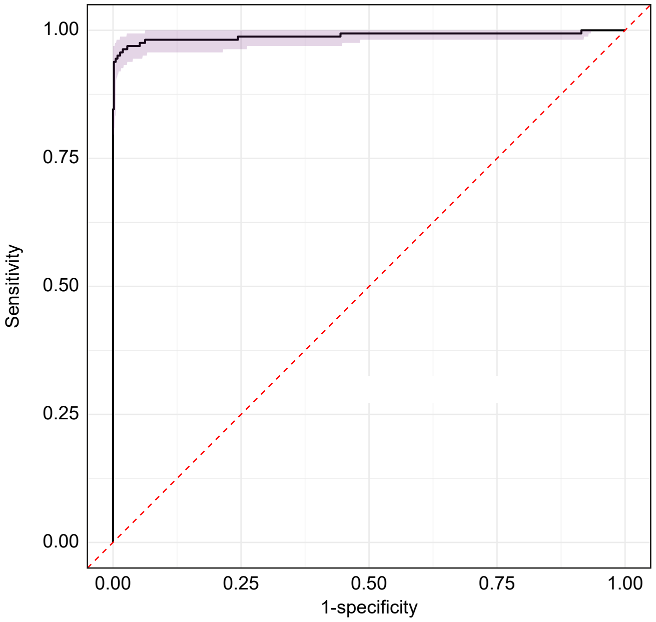

Fig. 2. ROC curve analysis of the total study population (n = 778) to assess diagnostic performance of REC for WD. ROC curve analysis was performed on the total study population to determine the Se, Sp, and predictive values of the REC. The area under the ROC curve was calculated, and the optimal cut-off value for the diagnosis of WD was determined by maximizing the Youden index. REC, relative exchangeable copper; ROC, receiver operating characteristic; Se, sensitivity; Sp, specificity; WD, Wilson disease.

observed in the overall study population (Table 3). In children <16-years old, the optimal REC threshold for diagnosing WD, as determined by the Youden index, was 14.03% with a Se of 95.8% (90.4–100%) and a Sp of 99.3% (97–100%) (Fig. 3). This threshold yielded a PPV of 99.1% (97.1–100%) and a NPV of 100%. In adults ≥16-years old, the optimal REC cut-off value was 14.1% with a Se of 96.8% (93.6–99.8%) and a Sp of 99.6% (96.7–100%) (Fig. 3B). The corresponding PPV was 94.4% (87.5–97.6%) and NPV was 99.7% (98.4–99.9%). In both age subgroups, previously published REC cut-offs of 15%[17] and 18.5%[13] also performed well for the diagnosis of WD (Table 3). Although both prior cut-offs achieved similarly high Sp (100%), Se was lower at 93.6% (89.3–96%) for the 15% cut-off and 87.7% (82.4–91%) for the 18.5% cut-off.

Finally, REC values were analyzed separately in the asymptomatic, hepatic, and hepatoneuropsychiatric forms of WD. The asymptomatic and hepatic forms showed significantly lower median REC values (26.6% and 31.3%, respectively) compared with the hepatoneuropsychiatric form (41%; p <0.0001).

The ability of REC to distinguish patients with symptomatic WD from those with asymptomatic WD was also evaluated. In this subanalysis, a REC threshold of 23.6% demonstrated the best diagnostic performance, yielding a Se of 79.7% but a low Sp of 48.4%. Notably, applying the 14% cut-off in this context resulted in a higher Se of 97.1%, but a markedly reduced Sp of 12.9%.

Overall, a REC cut-off of 14% demonstrated the best diagnostic performance across all age groups. Using this threshold, nine false negatives were identified, all of whom carried two pathogenic *ATP7B* variants (Table S4). These included five children (mean age 8 years), one of whom was asymptomatic and four had only mildly elevated transaminase levels. In addition, four adults (mean age 35 years) were misclassified, including three asymptomatic patients and one with hepatic involvement. All asymptomatic patients had no signs of liver involvement (*i.e.* normal liver tests, liver ultrasonography, and liver stiffness measurements) as well as no evidence of extrahepatic manifestations. All had total Cu, Cp, and CuEXC in the new ranges determined across all control samples. Only one false positive case was observed: a 21-year-old control individual who was later found to have a congenital disorder of glycosylation (Table S4). This patient presented with elevated transaminase levels. Total Cu and Cp levels were also markedly reduced, whereas 24-h UCE remained low (Table S4).

Discussion

Based on a large cohort of patients with WD, HTZ carriers, and controls, we confirmed that REC is a key biomarker for the diagnosis of WD and should be included in routine clinical practice as recommended by the new 2025 EASL-ERN Clinical Practice Guidelines on WD.[18] Measurement of CuEXC involves a straightforward ultrafiltration step followed by direct Cu quantification, yielding results as promptly as other Cu parameters. Importantly, CuEXC determination provides a rapid and reliable estimate of NCC, avoiding the issue of uninterpretable negative values often encountered with traditional NCC calculations. It also circumvents the use of more complex and time-consuming techniques, such as Cu speciation,[23–25] which are not easily adaptable to routine practice. In this large study involving 778 individuals, a REC threshold of 14% offered the optimal balance of Se and Sp for the diagnosis of WD across both pediatric and adult populations.

Compared with standard Cu markers, such as Cp and UCE, our findings confirm the superior diagnostic utility of REC in distinguishing healthy individuals from HTZ carriers and patients with WD, as previously reported.[17] In addition, REC outperformed CuEXC alone, which showed a lower Se (74.0%)

Table 3. Evaluation of REC cut-off for the diagnosis of WD according to age.

	REC 14%		REC 15%		REC 18.5%	
Characteristic	<16 years old*	≥16 years old†	<16 years old*	≥16 years old†	<16 years old*	≥16 years old†
Sensitivity (%)	95.8	96.8	92.1	94.8	88.2	89.7
Specificity (%)	99.3	99.6	100	100	100	100
PPV (%)	99.1	94.4	100	100	100	100
NPV (%)	100	99.7	96.2	97.9	94.4	95.8

NPV, negative predictive value; PPV, positive predictive value; REC, relative exchangeable copper.
*n = 91.
†n = 113.

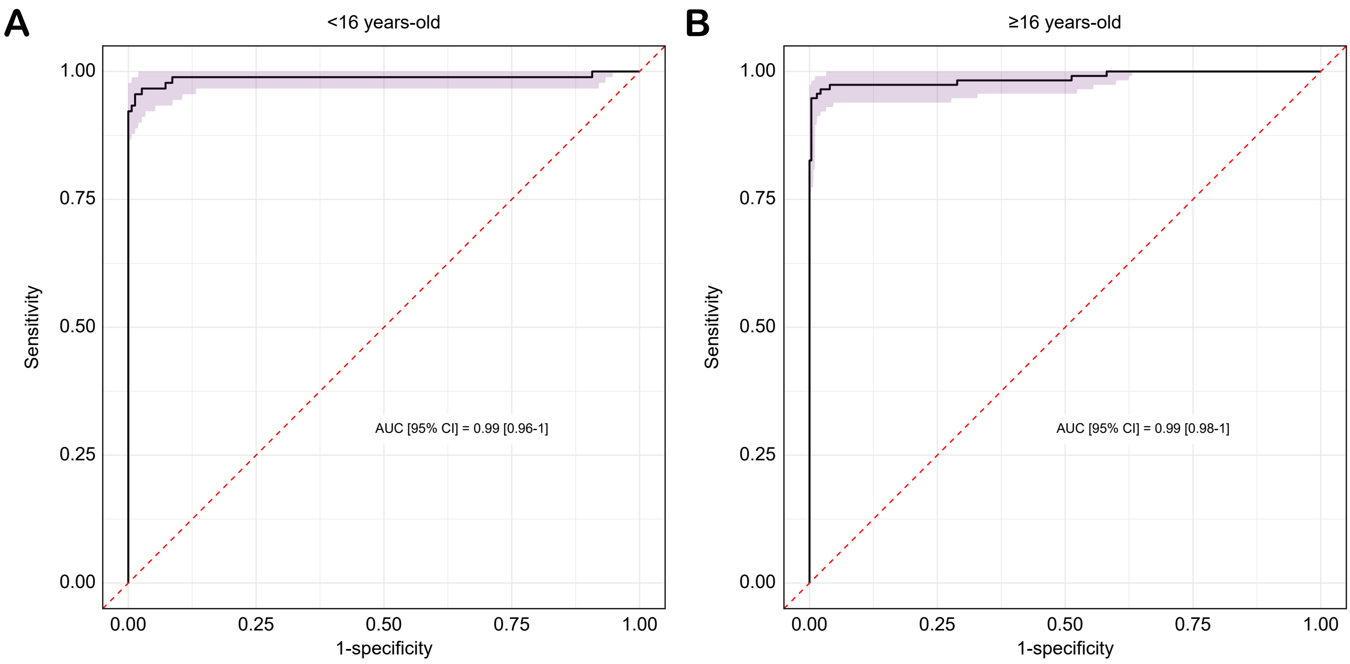

Fig. 3. ROC curves according to age. (A) Children <16-years old (n = 324); (B) adults ≥16-years old (n = 454). ROC curve analysis was performed on the total study population to determine the Se, Sp, and predictive values of the REC. The area under the ROC curve was calculated, and the optimal cut-off value for the diagnosis of WD was determined by maximizing the Youden index. REC, relative exchangeable copper; ROC, receiver operating characteristic; Se, sensitivity; Sp, specificity; WD, Wilson disease.

and Sp (90.8%) at the previously set cut-off of 1.15 μmol/L, corresponding to the upper limit of the reference range. In our study population, a Cp cut-off of <0.2 g/L, the threshold used in the Leipzig scoring system, demonstrated substantially lower Sp (81.9%) and PPV (67.6%) compared with REC, which achieved a Sp of 99.8% and a PPV of 99.1%. Cp levels can be normal or even elevated in patients with WD with significant liver inflammation and, conversely, can be decreased in patients with congenital disorders of glycosylation.[26] Similarly, the 24-h UCE cut-off of >0.6 μmol/24 h (above the upper normal value) showed limited Sp and Se. However, interpretation of these findings was hampered by substantial missing data, and the possibility that urine collection might not have been consistently performed correctly. Overall, these routine tests are major sources of false negative and false positive results.[8] Thus, the use of REC could help mitigate these limitations and improve diagnostic accuracy.

In our experience, assessing REC most often precluded the need for a liver biopsy to evaluate hepatic Cu levels in patients presenting with mild Cu abnormalities or carrying only one *ATP7B* variant. One notable case was an individual classified as a control, who presented with low total Cu and Cp levels but normal 24-h UCE, and a REC of 19.1%, who was later found to have a congenital disorder of glycosylation. By contrast, we identified a small subset of genetically confirmed WD cases with normal Cu metabolism markers and REC values below the 14% threshold. Specifically, five of 91 children (5.4%) and four of 113 adults (3.5%) with biallelic pathogenic *ATPB7* variants had a REC <14%. Among them, one child and three adults were asymptomatic with normal serum aminotransferase levels. These cases were identified through family screening of first-degree relatives with WD and underwent comprehensive evaluations that excluded both liver or neuropsychiatric involvement. This observation supports the concept of genetic WD, in which individuals carry a pathogenic genotype without manifestation of clinical symptoms, organ damage, or abnormalities in Cu metabolism.

These observations raise important considerations regarding the potential need for phenotype-adjusted REC thresholds. Although our data support the use of a universal 14% cut-off, which offers strong Se and Sp, a lower REC threshold might enhance diagnostic accuracy in asymptomatic individuals, particularly in the context of family screening. Although our current dataset lacks the statistical power to formally test this hypothesis, it warrants further investigation in longitudinal cohorts. Such studies could assess whether REC values rise over time in presymptomatic *ATP7B* variants carriers, potentially reflecting early or subclinical Cu accumulation. This dynamic perspective would shift the role of REC from a static diagnostic biomarker to a biological proxy for Cu toxicity and disease progression. If validated, REC could be instrumental in optimizing the timing of therapeutic interventions, especially for asymptomatic individuals with biallelic *ATP7B* variants. Finally, beyond its diagnostic value, REC could serve as a cost-effective triage tool within stepwise diagnostic algorithms, particularly in settings with limited access to molecular testing or liver biopsy. In such contexts, a normal REC could support a strategy of watchful waiting when no clinical signs or biochemical abnormalities are present. Conversely, an elevated REC should prompt expedited genetic confirmation and consideration of treatment initiation.

The inclusion of a large control group in this study enabled the establishment of reference ranges for both CuEXC and

REC. The recalculated reference range for CuEXC was 0.50–1.38 μmol/L (median 0.84 μmol/L; 5.3 μg/dl) and was slightly lower in children (median 0.77 μmol/L; 4.9 μg/dl) compared with adults. CuEXC levels were not influenced by sex. Although both studies used ICP-MS for copper measurement, Mariño *et al.* reported a narrower reference range (3.9–7.3 μg/dl) and lower median value (4.1 μg/dl) compared with those established in the present study.[14] This discrepancy is likely attributable to differences in study populations. In the study by Mariño *et al.*,[14] the control group included a high proportion of young individuals (53.6% of the 56 participants were children) as well as patients with minor gastrointestinal conditions or undergoing minor surgical interventions and voluntary adult participants without clinical or laboratory evidence of liver disease. To promote standardization in the literature, we recommend the use of the acronym CuEXC for exchangeable copper, as originally introduced by El Balkhi *et al.*,[10] rather than CuEx, as used by Mariño *et al.*[14]

Regarding REC values, children with lower CuEXC levels compared with adults and women and with higher total Cu levels than men had lower REC values compared with their respective counterparts. The observed sex-related differences in total Cu levels could be attributed to physiological hormonal factors, such as estrogen and progesterone secretion, as well as the use of contraceptives by women, as previously reported.[10] These findings suggest that both sex (with respect to total Cu) and age (with respect to CuEXC) should be considered in borderline diagnostic situations.

One of the main limitations of our study was its monocentric design, because all Cu balance assessments were conducted in a single laboratory in France affiliated with the reference center for WD until 2017. However, since then, the reference center in Lyon (France) has investigated the performance of REC in cases of acute liver failure and reported a diagnostic cut-off of 13.8% (Se 100% and Sp 99.6%) for distinguishing patients with newly diagnosed WD *vs.* those with non-Wilsonian hepatic diseases.[27] Subsequently, the methodology for CuEXC was developed in 10 other centers from French university hospitals and the accuracy and consistency of the measurements, including CuEXC, Cp, total Cu, and UCE, were ensured through participation in external quality assessment exercises. The reliability of the ultrafiltration method used to isolate CuEXC has been debated, notably by Solovyev *et al.*[28] and Del Castillo Busto *et al.*,[23] who raised concerns about variability stemming from differences in sample preparation and handling. To address these concerns, we followed a rigorously standardized protocol, including a precisely timed incubation step and validated equipment to minimize procedural variability. In addition, analysis of CuEXC values across two time periods revealed only a slight decrease in control group values after 2015, likely a result of the increased sample size. Importantly, REC values remained stable over time, supporting the robustness of the assay. Furthermore, interlaboratory quality control exercises conducted in collaboration with international centers (Spain, Canada, and Denmark) confirmed the reproducibility of CuEXC measurements, which is essential for the accurate calculation of REC.[14,29] Notably, an optimal REC cut-off value of 13.8% (Se 100% and Sp 99.6%),similar to the one identified in our study, was recently reported for the diagnosis of WD in pooled cohorts from Denmark and Spain.[30]

Accurate NCC (ANCC) assessment using Cu speciation and the relative ANCC (defined as the percentage of total Cu present as ANCC) have been reported as promising diagnostic and monitoring biomarkers for WD.[25] Ott *et al.*[24] compared different NCC measurement methodologies using either protein speciation (NCC-speciation) or exchangeable copper (NCC-Ex). Although the analysis was conducted in patients with clinically stable treated WD, NCC-Ex demonstrated a significant positive correlation with NCC-speciation ($r^2 = 0.66$, $p < 0.001$).

In addition, a dual filtration-based method for determining serum labile bound copper (LBC) and the LBC fraction (LBC/total Cu) was recently introduced; however, this approach requires further validation before it can be established as a reliable diagnostic tool.[31]

In conclusion, this large cohort study demonstrates that REC is a highly sensitive and specific biomarker for the diagnosis of WD. REC assessment provides a rapid and accurate diagnostic performance, particularly in individuals presenting with mild abnormalities in Cu metabolism. Our findings validate the integration of REC into clinical practice and reinforce its inclusion as a key biomarker in the diagnostic algorithm for WD, as proposed by the new EASL-ERN Clinical practice WD guidelines.[7] Incorporating REC into routine evaluation could facilitate earlier diagnosis and timely initiation of treatment, thereby helping to prevent irreversible tissue damage and improve quality of life for patients with WD. Ongoing longitudinal studies will further investigate the dynamics of REC and CuEXC during treatment and assess their correlation with conventional serum and urinary Cu biomarkers. These studies are expected to provide valuable insights into the prognostic value and therapeutic monitoring potential of both REC and CuEXC.

Affiliations

[1]*National Reference Centre for Wilson's Disease and Other Copper-Related Rare Diseases, Rothschild Foundation Hospital, Filière G2M, MetabERN, Paris, France;* [2]*Toxicology Department, Lariboisière University Hospital, AP-HP, Paris, France;* [3]*Department of Neurology, Rothschild Foundation Hospital, Paris, France;* [4]*Service de Médecine Génomique des Maladies Rares, Hôpital Universitaire Necker-Enfants Malades, INSERM 1163, Université Paris-Cité, Paris, France*

Abbreviations

ANCC, accurate NCC; Cp, ceruloplasmin; Cu, copper; CuEXC, exchangeable copper; HTZ, heterozygous carriers; ICP-MS, inductively coupled plasma-mass spectrometry; KFR Kayser–Fleischer rings; LBC, serum labile bound copper; MLPA, multiplex ligation-dependent probe amplification; NCC, non-ceruloplasmin-bound copper; NPV, negative predictive value; PPV, positive predictive value; REC, relative exchangeable copper; ROC, receiver operating characteristic; Se, sensitivity; Sp, specificity; Total Cu, total serum copper; UCE, urinary Cu excretion; WD, Wilson disease.

Financial support

The authors did not receive any financial support to produce this manuscript.

Conflicts of interest

AP is an adviser for Orphalan, Alexion, Vivet, and Univar and received institutional grants from Orphalan, Alexion, and AddMedica; ND-O is an adviser for Orphalan and Alexion. DD is an adviser for Orphalan and Alexion. CD is a consultant for TEVA Santé and received grants from TEVA Santé, Merz Pharma, ISIS Parkinson and ASDIA. MAO is an adviser for Orphalan. DR, CC, and JP have no conflicts of interest to declare.

Please refer to the accompanying ICMJE disclosure forms for further details.

Authors' contributions

Conception and design of the study: ND-O, CD, DD, and AP
Acquisition, analysis, or interpretation of the data: All authors
Drafting of the manuscript: ND-O, CD, DD, and AP
Revision of the manuscript: All authors.

Data availability

The datasets generated and analyzed during the current study are not publicly available due to the presence of potentially identifiable patient information. However, they are available from the corresponding author on reasonable request.

Acknowledgements

We thank Laura Smales for proofreading the manuscript.

Supplementary data

Supplementary data to this article can be found online at https://doi.org/10.1016/j.jhepr.2025.101537.

References

[1] Burkhead JL, Gray LW, Lutsenko S. Systems biology approach to Wilson's disease. Biometals 2011;24:455–466.
[2] Czlonkowska A, Litwin T, Dusek P, et al. Wilson disease. Nat Rev Dis Primers 2018;4:21.
[3] Poujois A, Mikol J, Woimant F. Wilson disease: brain pathology. Handb Clin Neurol 2017;142:77–89.
[4] Poujois A, Woimant F. Wilson's disease: a 2017 update. Clin Res Hepatol Gastroenterol 2018;42:512–520.
[5] Walshe JM. Cause of death in Wilson disease. Mov Disord 2007;22:2216–2220.
[6] Członkowska A, Niewada M, Litwin T, et al. Seven decades of clinical experience with Wilson's disease: report from the national reference centre in Poland. Eur J Neurol 2022;31:e15646.
[7] Schilsky ML, Roberts EA, Bronstein JM, et al. A multidisciplinary approach to the diagnosis and management of Wilson disease: executive summary of the 2022 Practice Guidance on Wilson disease from the American Association for the Study of Liver Diseases. Hepatology 2023;77:1428–1455.
[8] European Association for the Study of the Liver. EASL clinical practice guidelines: Wilson's disease. J Hepatol 2012;56:671–685.
[9] Danzeisen R, Araya M, Harrison B, et al. How reliable and robust are current biomarkers for copper status? Br J Nutr 2007;98:676–683.
[10] El Balkhi S, Poupon J, Trocello J-M, et al. Determination of ultrafiltrable and exchangeable copper in plasma: stability and reference values in healthy subjects. Anal Bioanal Chem 2009;394:1477–1484.
[11] Twomey PJ, Viljoen A, House IM, et al. Limitations of non-ceruloplasmin-bound copper in routine clinical practice. Gut 2007;56:154.
[12] Woimant F, Djebrani-Oussedik N, Poujois A. New tools for Wilson's disease diagnosis: exchangeable copper fraction. Ann Transl Med 2019;7:S70.
[13] El Balkhi S, Trocello J-M, Poupon J, et al. Relative exchangeable copper: a new highly sensitive and highly specific biomarker for Wilson's disease diagnosis. Clin Chim Acta 2011;412:2254–2260.
[14] Mariño Z, Molera-Busoms C, Badenas C, et al. Benefits of using exchangeable copper and the ratio of exchangeable copper in a real-world cohort of patients with Wilson disease. J Inherit Metab Dis 2023;46:982–991.
[15] Venelinov TI, Davies IM, Beattie JH. Dialysis-Chelex method for determination of exchangeable copper in human plasma. Anal Bioanal Chem 2004;379:777–780.
[16] Guillaud O, Brunet A-S, Mallet I, et al. Relative exchangeable copper: a valuable tool for the diagnosis of Wilson disease. Liver Int 2018;38:350–357.
[17] Trocello J-M, El Balkhi S, Woimant F, et al. Relative exchangeable copper: a promising tool for family screening in Wilson disease. Mov Disord 2014;29:558–562.
[18] European Association for the Study of the Liver. EASL-ERN clinical practice guidelines on Wilson's disease. J Hepatol 2025;82:690–728.
[19] Couchonnal E, Bouchard S, Sandahl TD, et al. ATP7B variant spectrum in a French pediatric Wilson disease cohort. Eur J Med Genet 2021;64:104305.
[20] Kumar M, Gaharwar U, Paul S, et al. WilsonGen a comprehensive clinically annotated genomic variant resource for Wilson's Disease. Sci Rep 2020;10:9037.
[21] Richards S, Aziz N, Bale S, et al. Standards and guidelines for the interpretation of sequence variants: a joint consensus recommendation of the American College of Medical genetics and Genomics and the Association for Molecular Pathology. Genet Med 2015;17:405–424.
[22] Walshe JM. Clinical Investigations Standing Committee of the Association of Clinical Biochemists. Wilson's disease: the importance of measuring serum caeruloplasmin non-immunologically. Ann Clin Biochem 2003;40:115–121.
[23] Del Castillo Busto ME, Cuello-Nunez S, Ward-Deitrich C, et al. A fit-for-purpose copper speciation method for the determination of exchangeable copper relevant to Wilson's disease. Anal Bioanal Chem 2022;414:561–573.
[24] Ott P, Sandahl T, Ala A, et al. Non-ceruloplasmin copper and urinary copper in clinically stable Wilson disease: alignment with recommended targets. JHEP Rep 2024;6:101115.
[25] Harrington CF, Carpenter G, Coverdale JPC, et al. Accurate non-ceruloplasmin bound copper: a new biomarker for the assessment and monitoring of Wilson disease patients using HPLC coupled to ICP-MS/MS. Clin Chem Lab Med (CCLM) 2025;63:320–328.
[26] Girard M, Poujois A, Fabre M, et al. CCDC115-CDG: a new rare and misleading inherited cause of liver disease. Mol Genet Metab 2018;124:228–235.
[27] Spirea D, Vanlemmens C, Parant F, et al. Performance of relative exchangeable copper for the diagnosis of Wilson Disease in acute liver failure. J Inherit Metab Dis 2025;48:e70024.
[28] Solovyev N, Ala A, Schilsky M, et al. Biomedical copper speciation in relation to Wilson's disease using strong anion exchange chromatography coupled to triple quadrupole inductively coupled plasma mass spectrometry. Analytica Chim Acta 2020;1098:27–36.
[29] Ott P, Ala A, Askari FK, et al. Designing clinical trials in Wilson's Disease. Hepatology 2021;74:3460–3471.
[30] Lorenzen C, Dons K, García-Solà C, et al. Relative exchangeable copper, exchangeable copper and total copper in the diagnosis of Wilson Disease. Liver Int 2025;45:e70089.
[31] Bornhorst JA, Bitzer AC, Day PL, et al. Total copper and labile bound copper fraction as a selective and sensitive tool in the evaluation of Wilson Disease. J Appl Lab Med 2024;9:1014–1027.

Keywords: Wilson disease; Metabolic disease; Exchangeable copper; Relative exchangeable copper; Non-ceruloplasmin-bound copper (NCC); Hepatolenticular degeneration.

Received 5 January 2025; received in revised form 3 July 2025; accepted 22 July 2025; Available online 31 July 2025

Journal of Hepatology, Volume *7*

Supplemental information

Relative exchangeable copper: A highly specific and sensitive biomarker for Wilson disease diagnosis

Nouzha Djebrani-Oussedik, Clément Desjardins, Mickaël Alexandre Obadia, Djamila Rahli, Corinne Collet, France Woimant, Joël Poupon, Dominique Debray, and Aurélia Poujois

Relative exchangeable copper: A highly specific and sensitive biomarker for Wilson disease diagnosis

Nouzha Djebrani-Oussedik, Clément Desjardins, Mickaël Alexandre Obadia, Djamila Rahli, Corinne Collet, France Woimant, Joël Poupon, Dominique Debray, Aurélia Poujois

Table of contents

Supplementary table legends……………………………………………………………2

Table S1……………………………………………………………………………………3

Table S2…………………………………………………………………………………...27

Table S3…………………………………………………………………………………...29

Table S4……………………………………………………………………………………31

Table S5…………………………………………………………………………………...33

Supplementary table legends:

Table S1. Genetic data of Wilson's Disease (WD) patients and Heterozygous carriers (HTZ).

Table S2. Results of copper metabolism assessment in controls, with comparisons by sex

Serum copper and CuEXC conversion: µmol/L × 6.3546 = µg/dL. F: female; M: male.

Table S3. Results of copper metabolism assessment in controls, with comparisons across age groups.

Serum copper and CuEXC conversion: µmol/L × 6.3546 = µg/dL. F: female; M: male.

Table S4. Main clinical, biological and genetic features associated with false negative and false positive REC results. F: Female; M: Male; Total Cu: Total serum copper; CuEXC: Exchangeable copper; REC: Relative exchangeable copper; Cp: Ceruloplasmin; 24 hr-UCE: 24-hour urinary copper excrection; AST: Aspartate aminotransferase; ALT: Alanine aminotransferase; PT: Prothrombin rate; KFR: Kayser Fleischer Ring; CDG: Congenital Disorder of Glycosylation.

Reference ranges were determined across all control samples

[1] Reference range: 8.5-28.2 µmol/L (24-179.2 µg/dL)

[2] Reference range: 0.50-1.38 µmol/L (3.2-8.8 µg/dL)

[3] Reference range: 2.6-9.5%

[4] Reference range: 0.17-0.4 g/L

[5] Reference range: 0.03-1.679 µmol/24h

Table S5. Comparison of total copper, exchangeable copper (CuEXC), and relative exchangeable copper (REC) levels in controls and heterozygous carriers (HTZ) across two time periods (2009–2014 vs. after 2015).

Table S1. Genetic data of (A.) Wilson Disease (WD) patients and (B.) Heterozygous carriers (HTZ)

A. **Variants in the 204 WD patients**

ALLELE 1	ALLELE 2
**Exon 2 : c.676C>T, p.Arg226Trp**<br>**Exon 8: c.2297C>G, p.Thr766Arg**	**Exon 2 : c.676C>T, p.Arg226Trp**<br>**Exon 8: c.2297C>G, p.Thr766Arg**
**Exon 2 : c.676C>T, p.Arg226Trp**<br>**Exon 8: c.2297C>G, p.Thr766Arg**	**Exon 2 : c.676C>T, p.Arg226Trp**<br>**Exon 8: c.2297C>G, p.Thr766Arg**
**Exon 3 : c.804dupT, p.Leu269Serfs*14**<br>**Exon 21: c.4135C>T, p.Pro1379Ser**	**Exon 18 : c.3859G>A, p.Gly1287Ser**
**Exon 2 : c.676C>T, p.Arg226Trp**<br>**Exon 16: c.3556G>A, p.Gly1186Ser**	**Exon 8 : c.2297C>G, p.Thr766Arg**
**Exon 1 : c.19_20delCA, p.Gln7Aspfs*14**<br>**Exon 18: c.3809A>G, p.Asn1270Ser**	**Exon 14 : c.3207C>A, p.His1069Gln**
**Exon 6 : c.1877G>C, p.Gly626Ala**	**Exon 6 : c.1877G>C, p.Gly626Ala**<br>**Exon 16 : c.3443T>C, p.Ile1148Thr**
**Intron 2 : c.1285+1G>A, p.?**	**Exon 8: c.2332>T, p.Arg778Trp**<br>**Exon 16 c.3451C>T, p.Arg1151Cys**
**Exon 2 : c.804dupT, p.Lys269Ter**<br>**Exon 21 : c.4135C>T, p.Pro1379Ser**	**Exon 18 : c.3859G>A, p.Gly1287Ser**
**Exon 2 : c.676C>T, p.Arg226Trp**	**Exon 6 : c.1877G>C, p.Gly626Ala**<br>**Exon 8 : c.2297C>G, p.Thr766Arg**
**Intron 18 : c.3904-2A>G, p.Val1302Glyfs*8**	**Exons 1-7: c.52-?_1285+?del**
**Exon 13 : c.3007G>A, p.Ala1003Thr**	**Exon14 : c.3106G>A, p.Val1036Ile**
**Exon 8 : c.2293G>A, p.Asp765Asn**	**Exon 8 : c.2293G>A, p.Asp765Asn**
**Exon 2 : c.122A>G, p.Asn41Ser**	**Exon 15 : c.3402del, p.Ala1135Glnfs*13**

**Exon 17 : c.3694 A>C, p.Thr1232Pro**	**Exon 17 : c.3694 A>C, p.Thr1232Pro**
**Exon 6 : c.1924G>C, p.Asp642His**	**Exon 8 : c.2131G>C, p.Gly711Arg**
**Exon 10 : c2558A>G, p.Asp853Gly**	**Exon 14 : c.3115G>T, p.Val1039Phe**
**Exon 10 : c2558A>G, p.Asp853Gly**	**Exon 14 : c.3115G>T, p.Val1039Phe**
**Exon 14 : c.3182G>A, p.Gly1061Glu**	**Exon 19 : c.3971A>G, p.Asn1324Ser**
**Exon 14 : c.3182G>A, p.Gly1061Glu**	**Exon 19 : c.3971A>G, p.Asn1324Ser**
**Exon 16 : c.3509delC, p.Thr1170Lysfs*5**	**Exon 18 : c.3843dupT, p.Val1282Cysfs*22**
**Intron 5 : c.1708-1G>A, p.?**	**Intron 5 : c.1708-1G>A, p.?**
**Intron 5 : c.1708-1G>A, p.?**	**Intron 5 : c.1708-1G>A, p.?**
**Intron 5 : c.1708-1G>A, p.?**	**Intron 5 : c.1708-1G>A, p.?**
**Intron 1 : c.51+4A>T, p.?**	**Exon 18 : c.3796G>A, p.Gly1266Arg**
**Exon 13 : c.2963G>T, p.Gly988Val**	**Exon 18 : c.3809A>G, p.Asn1270Ser**
**Exon 10 : c.2507G>A, p.Gly836Glu**	**Exon 10 : c.2507G>A, p.Gly836Glu**
**Exon 15 : c.3293C>G, p.Pro1098Arg**	**Exon 15 : c.3293C>G, p.Pro1098Arg**
**Exon 15 : c.3293C>G, p.Pro1098Arg**	**Exon 15 : c.3293C>G, p.Pro1098Arg**
**Intron 1 : c.51+4A>T, p.?**	**Intron 1 : c.51+4A>T, p.?**
**Intron 1 : c.51+4A>T, p.?**	**Intron 1 : c.51+4A>T, p.?**
**Intron 1 : c.51+4A>T, p.?**	**Intron 1 : c.51+4A>T, p.?**
**Intron 4 : c.1708-1G>A, p.?**	**Exon 17 : c.3620A>G, p.His1207Arg**
**Intron 8 : c.2122-8T>G, p.?**	**Exon 13 : c.2297C>T, p.Thr766Met**
**Exon 7 : c.1995G>A, p.Met665Ile**	**Exon 16 : c.3445G>A, p.Gly1149Arg**
**Exon 16 : c.3505A>G, p.Met1169Val**	**Exon 17 : c.3659C>T, p.Thr1220Met**
**Exon 20 : c.4112T>C, p.Leu1371Pro**	**Exon 20 : c.4112T>C, p.Leu1371Pro**
**Exon 14 : c.3207C>A, p.His1069Gln**	**Exon 14 : c.3207C>A, p.His1069Gln**
**Exon 8 : c.2128G>A, p.Gly710Ser**	**Exon 14 : c.3207C>A, p.His1069Gln**

Exon 14 : c.3207C>A, p.His1069Gln	Intron 15 : c.3556+1G>T, p.Arg1186Glufs*10
Exon 14 : c.3207C>A, p.His1069Gln	Intron 15 : c.3556+1G>T, p.Arg1186Glufs*10
Exon 10 : c.2507G>A, p.Gly836Glu	Unidentified
Exon 8 : c.2333G>A, p.Arg778Gln	Exon 14: c.3207C>A, p.His1069Gln
Exon 13 : c.3059A>G, p.Lys1020Arg	Exon 13: c.3059A>G, p.Lys1020Arg
Exon 4 : c.1543+1_1544-1_1707+1_1708-1del, p.?	Exon 2 : c.388C>T, p.Arg130Cys
Exon 14 : c.3207C>A, p.His1069Gln	Exon 4 : deletion
Exon 8 : c.2297C>G, p.Thr766Arg	Exon 8 : c.2332C>T, p.Arg778Trp
Exon 18 : c.3799G>A, p.Asp1267Asn	Intron 4 : c.1708-34G>A, p.?
Intron 10 : c.1869+2T>C, p.Ser623Alafs*23	Exon 10 : c.2575G>A, p.Gly859Arg
Exon 20 : c.4092_4093del, p.Ser1365Cysfs*12	Intron 20 : c.4125-1G>T, p.?
Exon 20 : c.4092_4093del, p.Ser1365Cysfs*12	Intron 20 : c.4125-1G>T, p.?
Intron 4 : c.708-1G>A, p.?	Exon 6 : c.1934T>G, p.Met645Arg
Intron 4 : c.708-1G>A, p.?	Intron 4 : c.708-1G>A, p.?
Exon 2 : c.347T>C, p.Ile116Thr	Exon 13 : c.2930C>T, p.Thr977Met
Exon 14 : c.3207C>A, p.His1069Gln	Exon 15 : c.3263T>A, p.Leu1088*
Intron 1 : c.51+4A>T, p.?	Exon 17: c.3694A>C, p.Thr1232Pro
Exon 12 : c.2795C>A, p.Ser932Ter	Exon 12: c.2795C>A, p.Ser932Ter
Exon 12 : c.2795C>A, p.Ser932Ter	Exon 12 : c.2795C>A, p.Ser932Ter
Intron 4 : c.1707+2dupT	Intron 4 : c.1707+2dupT
Intron 4 : c.1707+2dupT	Intron 4 : c.1707+2dupT
Exon 8 : c.2297C>G, p.Thr766Arg	Exon 14 : c.3207C>A, p.His1069Gln
Exon 14 : c.3207C>A, p.His1069Gln	Exon 14 : c.3207C>A, p.His1069Gln

**Exon 8 : c.2128G>A, p.Gly710Ser**	**Exon 16 : c.3505A>G, p.Met1169Val**
**Exon 8 : c.2303C>T, p.Pro768Leu**	**Exon 20 : c.4092_4093del, p.Ser1365Cysfs*12**
**Exon 14 : c.3207C>A, p.His1069Gln**	**Exon 14 : c.3207C>A, p.His1069Gln**
**Exon 16 : c.3443T>C, p.Ile1148Thr**	**Exon 18 : c.3818C>T, p.Pro1273Leu**
**Exon 12 : c.2804C>T, p.Thr935Met**	**Exon 12 : c.2804C>T, p.Thr935Met**
**Exon 12 : c.2804C>T, p.Thr935Met**	**Exon 12 : c.2804C>T, p.Thr935Met**
**Exon 12 : c.2804C>T, p.Thr935Met**	**Exon 12 : c.2804C>T, p.Thr935Met**
**Exon 4 : c.1705_1707-10del, p.Thr569del**	**Exon 5 : c.1745_1746del, p.Ile582Argfs*25**
**Exon 10 : c.2532delA, pVal845 Serfs*28**	**Exon 18 : c.3821C>A, p.Ala1274Asp**
**Exon 10 : c.2532delA, pVal845 Serfs*28**	**Exon 18 : c.3821C>A, p.Ala1274Asp**
**Exon 10 : c.2532delA, pVal845 Serfs*28**	**Exon 18 : c.3821C>A, p.Ala1274Asp**
**Exon 14 : c.3207C>A, p.His1069Gln**	**Exon 17 : c.3646G>A, p.Val1216Met**
**Exon 2 : c.331C>T, p.Gln111***	**Exon 7 : c.1995G>A, p.Met665Ile**
**Exon 8 : c.2305A>G, p.Met769Val**	**Exon 11 : c.2605G>T, p.Gly869Ter**
**Exon 8 : c.2304dup, p.Met769Hisfs*26**	**Exon 8 : c.2304dup, p.Met769Hisfs*26**
**Exon 6 : c.1774A>T, p.Ile592Phe**	**Exon 4 : c.1145C>G, p.Ser382Cys**
**Exon 14 : c.3207C>A, p.His1069Gln**	**Exon 14 : c.3207C>A, p.His1069Gln**
**Exon 14 : c.3083_3085delinsG>C, p.Lys1028Serfs*40**	**Exon 16 : c.3445G>C, p.Gly1149Arg**
**Exon 8 : c.2128G>A, p.Gly710Ser**	**Intron 19 : c.4124+6T>C, p.?**
**Exon 8 : c.2336G>A, p.Trp779Ter**	**Exon 14: c.3207C>A, p.His1069Gln**
**Exon 19 : c.3955C>T, p.Arg1319Ter**	**Exon 19 : c.3955C>T, p.Arg1319Ter**
**Intron 4 : c.-362C>T, p.?**	**5'UTR / prom: c.-362C>T, p.?**
**Exon 3 : c.958_961delinsAGTT, p.Pro320Serfs*6**	**Exon 3 : c.958_961delinsAGTT, p.Pro320Serfs*6**

**Intron 13 : c.3061-12T>A, p.?**	**Unidentified**
**Exon 8 : c.2128G>A, p.Gly710Ser**	**Exon 8 : c.2304dupC, p.Met769HisfsX26**
**Exon 5 : c.1708-1G>A, p.Glu570Alafs*13**	**Exon 5 : c.1708-1G>A, p.Glu570Alafs*13**
**Exon 5 : c.122del, p.Asn41fs**	**Intron 12 : c.2576-2A>G, p.Lys859Argfs*10**
**Intron 1 : c.51+4A>T, p.?**	**Exon 8 : c.2128G>A, p.Gly710Ser**
**Exon 6 : c.1934T>G, p.Met645Arg**	**Exon 18 : c.3809A>G, p.Asn1270Ser**
**Exon 2 : c.778dupC, p.Gln260ProfsX10**	**Intron 10 : c.1869+5_1869+8del, p.?**
**Exon 15 : c.3263T>A, p.Leu1088***	**Intron 15 : c.3412+2T>C, p.?**
**Exon 11 : c.2605G>A, p.Gly869Arg**	**Intron 18 : c.3412+2T>C, p.Leu1138Profs*19**
**Exon 8 : c.2304dupC, p.Met769Hisfs*26**	**Exon 1 : c.3190G>A, p.Glu1064Lys**
**Exon 18 : c.3842G>A, p.Gly1281Asp**	**Intron 17 : c.3699+280A>G, p.?**
**Exon 7 : c.1995G>A, p.Met665IIe**	**Intron1 : c.51+4A>T, p.?**
**Exon 2 : c.865C>T, p.Gln289X**	**Exon 8 : c.232C>G, p.Arg778Gly**
**Exon 13 : c.3059A>G, p.Lys1020Arg**	**Exon 13 : c.3059A>G, p.Lys1020Arg**
**Exon 13 : c.3059A>G, p.Lys1020Arg**	**Exon 13 : c.3059A>G, p.Lys1020Arg**
**Exon 13 : c.3059A>G, p.Lys1020Arg**	**Exon 13 : c.3059A>G, p.Lys1020Arg**
**Exon 19 : c.3955C>T, p.Arg1319***	**Exon 19 : c.3955C>T, p.Arg1319***
**Intron 9 : c.2448-41G>A, p.?**	**Unidentified**
**Exon 13 : c.2930C>T, p.Thr977Met**	**Intron 13 : c.3061-1G>A, p.Gly1021Glufs*16**
**Intron 1 : c.51+4A>T, p.?**	**Intron 5 : c.1708-1G>C, p.?**
**Exon 2 : c.122A>G, p.Asn41Ser**	**Exon 18 : c.3743A>C, p.Lys1248Thr**
**Exon 2 : c.122A>G, p.Asn41Ser**	**Exon 18 : c.3743A>C, p.Lys1248Thr**
**Exon 18 : c.3809A>G, p.Asn1270Ser**	**Exon 2: insertion ? C.?, p.?**
**Exon 17 : c.3694 A>C, p.Thr1232Pro**	**Exon 17 : c.3694A>C, p.Trp1232Pro**
**Exon 1 : c.51+5G>A, p.?**	**Exon 10 : c.2549C>A, p.Thr850Asn**

Exon 19 : c.3971A>G, p.Asn1324Ser	Exon 20 : c.4040delG, p.Gly1347AlafsTer46
Exon 13 : c.3007G>A, p.Ala1003Thr	Exon 14 : c.3207C>A, p.His1069Gln
Exon 18 : c.3895C>T, p.Leu1299Phe	Exon 18 : c.3895C>T, p.Leu1299Phe
Exon 14 : c.3188C>T, p.Ala1063Val	Exon 18 : c.3743A>C, p.Lys1248Thr
Intron 1 : c.51+4A>T, p.?	Exon 8 : c.2237T>A, p.Val746Asp
Intron 1 : c.51+4A>T, p.?	Exon 8 : c.2237T>A, p.Val746Asp
Exon 12 : c.2804C>T, p.Thr935Met	Exon 12: c.2804C>T, p.Thr935Met
Exon 2 : c.1136del, p.Gly379Alafs*2	Exon 8 : c.2333G>T, p.Arg778Leu
Exon 11 : c.2621C>T, p.Ala874Val	Exon 14 : c.3182G>A, p.Gly1061Glu
Exon 14 : c.3207C>A, p.His1069Gln	Exon 15 : c.3402del, p.Ala1135GlnfsTer13
Exon 14 : c.3207C>A, p.His1069Gln	Exon 18 : c.3842G>A, p.Gly1281Asp
Exon 2 : c.505C>T, p.Gln169*	Exon 13 : c.2936T>G, p.Leu979Arg
Exon 6 : c.1934T>G p.Met645Arg	Exon 13 : c.2911G>C, p.Ala971Pro
Exon 14 : c.3207C>A, p.His1069Gln	Exon 16 : c.3547G>A, p.Ala1183Thr
Exon 21 : c.4144G>T p.Glu1382*	Exon 21 : c.4144G>T, p.Glu1382*
Exon 7 : c.2009_2015del, p.Tyr670_Met671delinsTer	Exon 13 : c.3035G>T, p.Gly1012Val
Exon 14 : c.3207C>A, p.His1069Gln	Unidentified
Exon 13 : c.2930C>T, p.Thr977Met	Exon 13 : c.2936T>A, p.Leu979Gln
Intron 10 : c.1869+5_1869+8delGTAA, p.?	Exon 19 : c.3914T>C, p.Leu1305Pro
Exon 16 : c.3461T>C, p.Leu1154Pro	Exon 18 : c.3842G>A, p.Gly1281Asp
Exon 13 : c.3053C>T; p.Ala1018Val	Exon 17 : c.3617_3618insA, p.His1207Alafs*52
No variant detected in 2012 (Sanger and MLPA)	No variant detected in 2012 (Sanger and MLPA)
Unidentified ~~No variant detected in 2012 (Sanger and MLPA)~~	Unidentified ~~No variant detected in 2012 (Sanger and MLPA)~~

Exon 13 : c.3059A>G, p.Lys1020Arg	Exon 13 : c.3059A>G, p.Lys1020Arg
Exon 7 : c.3659C<T, p.Thr1220Met	Exon 14 : c.3083-3085delinsGT, p.Lys1028Serfs*40
Exon 14 : c.3098C>T, p.Thr1033Ile	Exon 14 : c.3098C>T, p.Thr1033Ile
Exon 8 : c.2336G>A, p.Trp779Ter	Exon 14: c.3063A>G, p.Ile1021Met
Exon 13 : c.2912C>T, p.Ala971Val	Intron 16 : c.3556+1G>T, p.?
Exon 10 : c2519C>T, p.Pro840Leu	Exon 14: c.3104G>A, p.Gly1035Asp
Exon 13 : c.3059A>G, p.Lys1020Arg	Exon 13 : c.3059A>G, p.Lys1020Arg
Exon 8 : c.2304dupC, p.Met769Hisfs*26	Exon 8 : c.2304dupC, p.Met769Hisfs*26
Exon 2 : c.813C>A, p.Cys271*	Exon 8 : c.2145C>T, p.Tyr715Tyr
Exon 14 : c.3207C>A, p.His1069Gln	Exon 17 : c.3694A>C, p.Thr1232Pro
Exon 10 : c.2549C>T, p.Thr850Ile	Exon 18 : c.3818C>A, p.Pro1273Gln
Exon 10 : c.2549C>T, p.Thr850Ile	Exon 18 : c.3818C>A, p.Pro1273Gln
Exon 2 : c.314C>A, p.Ser105*	Exon 5 : c.1846C>T, p.Arg616Trp
Exon 6 : c.1934T>G, p.Met645Arg	Exon 6 : c.1934T>G, p.Met645Arg
Exon 6 : c.1934T>G, p.Met645Arg	Exon 6 : c.1934T>G, p.Met645Arg
Intron 1 : c.51+4A>T, p.?	Intron 1 : c.51+4A>T, p.?
Exon 14 : c.3207C>A, p.His1069Gln	Exon 14 : c.3207C>A, p.His1069Gln
Exon 10 : c.2532del:p.Val845Serfs*28	Exon 10 : c.2532del:p.Val845Serfs*28
Exon 8 : c.2292C>T, p.Phe764Phe	Exon 8 : c.2292C>T, p.Phe764Phe
Exon 2 : c.213_214delAT, p.Val73Glufs*4	Unidentified
Exon 14 : c.3207C>A, p.His1069Gln	Exon 18 : c.3809A>G, p.Asn1270Ser
Exon 4 : c.1705_1707+10del13 p.Thr569del	Exon 4 : c.1705_1707+10del13, p.Thr569del
Exon 14 : c.3207C>A, p.His1069Gln	Exon 14 : c.3207C>A, p.His1069Gln
Exon 2 : c.331C>T, p.Gln111Ter	Exon 19 : c.3914T>C, p.Leu1305Pro

**Exon 6 : c.1934T>G, p.Met645Arg**	**Unidentified**
**Exon 2 : c.670dup, p.Ile224Asnfs*2**	**Exon 7 : c.2046G>C, p.Met682Ile**
**Exon 14 : c.3207C>A, p.His1069Gln**	**Exon 19 : c.3914T>C, p.Leu1305Pro**
**Exon 8 : c.2332C>T, p.Arg778Trp**	**Exon 20 : c.4049T>G, p.Leu1350Arg**
**Exon 19 : c.4021G>A, p.Gly1341Ser**	**Unidentified**
**Exon 13 : c.2971A>G, p.Thr991Ala**	**Exon 18 : c.3872C>T, p.Ala1291Val**
**Exon 8 : c.2128G>A, p.Gly710Ser**	**Exon 8 : c.2305A>G, p.Met769Val**
**Exon 8 : c.2304dupC, p.Met769Hisfs*26**	**Exon 20 : c.4124G>C, p.Cys1375Ser**
**Exon 8 : c.2304dupC, p.Met769Hisfs*26**	**Exon 14 : c.3207C>A, p.His1069Gln**
**Exon 4 : c.1625T>C p.Ile542Thr**	**Intron 11 : c.2731-1G>A, p.?**
**Exon 12 : c.2753A>G, p.Asp918Gly**	**Exon 16 : c.3556G>A, p.Gly1186Ser**
**Exon 12 : c.2753A>G, p.Asp918Gly**	**Exon 16 : c.3556G>A, p.Gly1186Ser**
**Exon 8 : c.2128G>A, p.Gly710Ser**	**Exon 8 : c.2304dupC, p.Met769Hisfs*26**
**Exon 14 : c.3061-12T>A p.(?)**	**Exon 19 : c.3955C>T, p.(Arg1319Ter)**
**Exon 2 : c.213_214delAT, p.Val73Glufs*4**	**Exon 7 : c.1995G>A, p.Met665Ile**
**Exon 2 : c.213_214delAT, p.Val73Glufs*4**	**Exon 7 : c.1995G>A, p.Met665Ile**
**Intron 1 : c.51+4A>T, p.?**	**Exon 3 : c.1512dup, p.Asn505Ter**
**Exon 2 : c.721C>T, p.Gln241***	**Exon 2: c.721C>T, p.Gln241***
**Exon 8 : c.2305A>G, p.Met769Val**	**Exon 20 : c.4051C>T, Gln1351***
**Exon 8 : c.2305A>G, p.Met769Val**	**Exon 20 : c.4051C>T, Gln1351***
**Intron 1 : c.51+4A>T, p.?**	**Exon 10 : c.2512A>G; p.Lys838glu**
**Intron 1 : c.51+4A>T, p.?**	**Exon 10 : c.2512A>G; p.Lys838glu**
**Intron 2 : c.1285+1G>A, p.?**	**Exon 10 : c.2501T>A, p.Val834Asp**
**Exon 5 : c.1774A>T, p.IIe592Phe**	**Exon 10: c.2525A>G, p.Asp842Gly**
**Intron 1 : c.51+4A>T, p.?**	**Intron 1 : c.51+4A>T, p.?**

**Intron 1 : c.51+4A>T, p.?**	**Intron 1 : c.51+4A>T, p.?**
**Exon 13 : c.3059A>G, p.Lys1020Arg**	**Exon 14 : c.3207C>A, p.His1069Gln**
**Intron 7 : c.2122-8T>G, p.?**	**Exon 14 : c.3207C>A, p.His1069Gln**
**Exon 8 : c.2324C>T, p.Ala775Val**	**Exon 19 : c.3992A>G,p.Tyr1331Cys**
**Exon 8 : c.2324C>T, p.Ala775Val**	**Exon 19 : c.3992A>G,p.Tyr1331Cys**
**Exon 16 : c.3551T>C, p.Ile1184Thr**	**Intron 12 : c.2866-1521G>A, p.?**
**Exon 12 : c.2752G>A, p.Asp918Asn**	**Exon 12 : c.2752G>A p.Asp918Asn**
**Intron 12 : c.2865+1G>A, p.?**	**Exon 14 : c.3083A>G, p.Lys1028Arg**
**Exon 8 : c.2123T>C, p.Leu708Pro**	**Exon 14 c.3207C>A, p.His1069Gln**
**Exon 3 : c.1512dupT, p.Asn505***	**Exon 9 : c.2383C>T, p,Leu795Phe**
**Intron 11 : c.2731-2A>G, p.?**	**Exon 17 : c.3688A>G, p.(Ile1230Val)**
**Intron 1 : c.51+4A>T, p.?**	**Exon 14 : c.3207C>A, p.His1069Gln**
**Exon 12 : c.2762G>A, p.Ser921Asn**	**Unidentified**
**Exon 14 : c.3207C>A, p.His1069Gln**	**Exon 14 : c.3207C>A, p.His1069Gln**
**Exon 6 : c.1877G>C p.Gly626Ala**	**Exon 18 : c.3842G>A p.Gly 1281Asp**
**Exon 14 : c.3191A>C, p.Glu1064Ala**	**Exon 17 : c.3694A>C, p.Thr1232Pro**
**Exon 14 : c.3191A>C, p.Glu1064Ala**	**Exon 17 : c.3694A>C, p.Thr1232Pro**
**Exon 16 : c.3426G>C, p.Gln1142His**	**Exon 16 : c.3443T>C, p.Ile1148Thr**
**Exon 8 : c.2333G>T, p.Arg778Leu**	**Exon 18 : c.3809A>G, p.Asn1270Ser**
**Exon 18 : c.3817C>T, p.Pro1273Leu**	**Exon 18 : c.3817C>T, p.Pro1273Leu**
**Exon 14 : c.3207C>A, p.His1069Gln**	**Exon 14 : c.3207C>A, p.His1069Gln**
**Exon 14 : c.3207C>A, p.His1069Gln**	**Exon 14 : c.3207C>A, p.His1069Gln**
**Exon 13 : c.2930C>T, p.Thr977Met**	**Exon 13 : c.2930C>T, p.Thr977Met**

B. Variants in the 359 Heterozygous carriers (HTZ)

ALLELE 1
**Exon 14 : c.3207C>A, p.His1069Gln**
**Exon 13 : c.3007G>A, p.Ala1003Thr and Exon14: c.3106G>A, p.Val1036Ile**
**Exon 8 : c.2293G>A, p.Asp765Asn**
**Exon 19 : c.4004G>A, p.Gly1335Glu**
**Exon 19 : c.4004G>A, p.Gly1335Glu**
**Exon 8 : c.2304dupC, p.Met769Hisfs*26**
**Exon 2 : c.1136delG, p.Gly379Alafs*2**
**Exon 8 : c.2304dupC, p.Met769Hisfs*26**
**Exon 14 : c.3071_3072delTG, p.Val1024Aspfs*3**
**Exon 6 : c.1924G>C, p.Asp642His**
**Exon 8 : c.2131G>C, p.Gly711Arg**
**Exon 8 : c.2131G>A, p.Gly711Arg**
**Exon 10 : c.2507G>A, p.Gly836Glu**
**Exon 13 : c.2963G>T, p.Gly988Val**
**Exon 13 : c.2963G>T, p.Gly988Val**
**Exon 18 : c.3809A>G, p.Asn1270Ser**
**Exon 2 : c.576_577insG, p.Gln193Alafs*11**
**Exon 10 : c.2532del, p.Val845SerfsX28**
**Exon 2 : c.1063C>T, p.Gln355***
**Intron 1 : c.51+4A>T, p.?**
**Intron 1 : c.51+4A>T, p.?**
**Intron 1 : c.51+4A>T, p.?**
**Intron 1 : c.51+4A>T, p.?**
**Exon 14 : c.3207C>A, p.His1069Gln**

**Exon 14 : c.3207C>A, p.His1069Gln**
**Exon 14 : c.3188C>T, p.Ala1063Val**
**Exon 10 : c.2549C>T, p.Thr850Ile**
**Exon 15 : c.3263T>A, p.Ile1088Ter**
**Exon 15 : c.3263T>A, p.Ile1088Ter**
**Exon 15 : c.3263T>A, p.Ile1088Ter**
**Exon 15 : c.3263T>A, p.Ile1088Ter**
**Exon 7 : c.2009_2015del, pTyr670_Met671delinsTer**
**Exon 2 : c.865C>T, p.Gln289***
**Exon 14 : c.3207C>A, p.His1069Gln**
**Exon 2 : c.213_214del, p.Val73GlufsTer4**
**Exon 2 : c.213_214del, p.Val73GlufsTer4**
**Intron 1 : c.51+4A>T, p.?**
**Exon 14 : c.3207C>A, p.His1069Gln**
**Exon 14 : c.3207C>A, p.His1069Gln**
**Exon 13 : c.3059A>G, p.Lys1020Arg**
**Exon 13 : c.3059A>G, p.Lys1020Arg**
**Exon 13 : c.3059A>G, p.Lys1020Arg**
**Exon 13 : c.3059A>G, p.Lys1020Arg**
**Exon 13 : c.3059A>G, p.Lys1020Arg**
**Intron 5 : c.1869+2T>C, p.?**
**Intron 5 : c.1869+2T>C, p.?**
**Exon 8 : c.2303C>T, p.Pro768Leu**
**Exon 3 : c.1512dupT, p.Asn505***
**Exon 14 : c.3207C>A, p.His1069Gln**

**Exon 14 : c.3207C>A, p.His1069Gln**
**Intron 1 : c.51+4A>T, p.?**
**Intron 1 : c.51+4A>T, p.?**
**Exon 20 : c.4092_4093delGT, p.Ser1365Lysfs*12**
**Exon 20 : c.4092_4093delGT, p.Ser1365Lysfs*12**
**Exon 20 : c.4092_4093delGT, p.Ser1365Lysfs*12**
**Exon 20 : c.4092_4093delGT, p.Ser1365Lysfs*12**
**Exon 18 : c.3809A>G, p.Asn1270Ser**
**Exon 18 : c.3809A>G, p.Asn1270Ser**
**Exon 18 : c.3809A>G, p.Asn1270Ser**
**Exon 19 : c.3955C>T, p.Arg1319***
**Exon 14 : c.3207C>A, p.His1069Gln**
**Exon 8 : c.2128G>A, p.Gly710Ser**
**Exon 8 : c.2332C>G, p.Arg778Gly**
**Exon 18 : c.3799G>A p.Asp1267Asn**
**intron 4: c.1708G>A, p.?**
**Exon 21 : c.4144G>T, p.Glu1382***
**Exon 21 : c.4144G>T, p.Glu1382***
**Exon 21 : c.4144G>T, p.Glu1382***
**Exon 14 : c.3207C>A, p.His1069Gln**
**Exon 16 : c.3532A>G, p.Thr1178Ala**
**Exon 18 : c.3799G>A p.Asp1267Asn**
**Exon 18 : c.3809A>G, p.Asn1270Ser**
**Exon 2 : c.331C>T, p.Gln111***
**Exon 20 : c.4092_4093del, p.Ser1365Cysfs*12**

**Intron 21 : c.4125-1G>T, p.?**
**Exon 18 : c.3809A>G, p.Asn1270Ser**
**Exon 14 : c.3207C>A, p.His1069Gln**
**Exon 19 : c.3965G>C p.Arg1322Pro**
**Exon 19 : c.3965G>C p.Arg1322Pro**
**Intron 4 : c.1707+2dupT, p.?**
**Intron 4 : c.1707+2dupT, p.?**
**Intron 4 : c.1707+2dupT, p.?**
**Exon 14 : c.3207C>A, p.His1069Gln**
**Exon 10 : c.2501T>A, p.Val834Asp**
**Exon 20 : c.4092_4093delGT, p.Ser1365Lysfs*12**
**Exon 20 : c.4092_4093del, p.Ser1365Cysfs*12**
**Exon 6 : c.3193G>C, p.Ala1065Pro**
**Intron 12 : c.2731-2A>G, p.Ser911Argfs*15**
**Intron 12 : c.2731-2A>G, p.Ser911Argfs*15**
**Exon 13 : c.3007G>A, p.Ala1003Thr**
**Exon 10 : c.2930C>T, p.Thr977Met**
**Exon 3 : c.1512dupT, p.Asn505***
**Exon 20 : c.4092_4093del, p.Ser1365Cysfs*12**
**Exon 13 : c.3057_3059del, p.His1019_Lys1020delinsGln**
**Exon 8 : c.2267C>T, p.Ala756Val**
**Exon 21 : c.4261C>T, p.Gln1421***
**Exon 16 : c.3443T>C, p.Ile1148Thr**
**Exon 18 : c.3818C>T, p.Pro1273Leu**
**Exon 13 : c.2930C>T, p.Thr977Met**

**Exon 21 : c.4261C>T, p.Gln1421***
**Exon 17 : c.3694A>C, p.Thr1232Pro**
**Exon 2 : c.676C>T,p.Arg226Trp and Exon 8 : c.2297C>G, p.Thr766Arg**
**Exon 2 : c.676C>T,p.Arg226Trp and Exon 8 : c.2297C>G, p.Thr766Arg**
**Exon 2 : c.676C>T,p.Arg226Trp and Exon 8 : c.2297C>G, p.Thr766Arg**
**Exon 2 : c.676C>T,p.Arg226Trp and Exon 8 : c.2297C>G, p.Thr766Arg**
**Exon 14 : c.865C>T, p.Gln289***
**Exon 19 : c.3974delT, p.Leu1325Argfs*5**
**Exon 19 : c.3974delT, p.Leu1325Argfs*5**
**Exon 19 : c.3974del p.Leu1325ArgfsTer5**
**Exon 19 : c.3974del p.Leu1325ArgfsTer5**
**Exon 14 : c.3207C>A, p.His1069Gln**
**Intron 7 : c.1946+5G>A, p.?**
**Exon 14 : c.3207C>A, p.His1069Gln**
**Exon 20 : c.4092_4093delGT, p.Ser1365Lysfs*12**
**Exon 20 : c.4092_4093del, p.Ser1365Cysfs*12**
**Exon 16 : c.3445G>C, p.Gly1149Arg**
**Exon 14 : c.3207C>A, p.His1069Gln**
**Exon 14 : c.3207C>A, p.His1069Gln**
**Intron 20 : c.4124+6T, p.?**
**Exon 14 : c.3207C>A, p.His1069Gln**
**Exon 14 : c.3207C>A, p.His1069Gln**
**Exon 19 : c.3955C>T, p.Arg1319***
**Intron 16 : c.3556+3A>G, p.?**
**Exon 16 : c.3547G>A, p.Ala1183Thr**

**Exon 8 : c.2128G>A, p.Gly710Ser**
**Exon 8 : c.2304dupC, p.Met769Hisfs*26**
**Exon 8 : c.2336G>A, p.Trp779***
**Exon 10 : c.2507G>A, p.Gly836Glu**
**Exon 14 : c.3207C>A, p.His1069Gln**
**Exon 2 : c.676C>T,p.Arg226Trp and Exon 8 : c.2297C>G, p.Thr766Arg**
**Exon 10 : c.2507G>A, p.Gly836Glu**
**Exon 10 : c.2507G>A, p.Gly836Glu**
**Exon 10 : c.2507G>A, p.Gly836Glu**
**Exon 10 : c.2507G>A, p.Gly836Glu**
**Exon 14 : c.3207C>A, p.His1069Gln**
**Exon 14 : c.3207C>A, p.His1069Gln**
**Exon 8 : c.2207C>T, p.Ala736Val**
**Exon 18 : c.3842G>A, p.Gly1281Asp**
**Exon 18 : c.3842G>A, p.Gly1281Asp**
**Exon 11 : c.2605G>A, p.Gly869Arg**
**Intron 15 : c.3412+2T>C, p.?**
**Intron 15 : c.3412+2T>C, p.?**
**Intron 15 : c.3412+2T>C, p.?**
**Exon 14 : c.3190G>A, p.Glu1064Lys**
**Exon 14 : c.3188C>T, p.Ala1063Val**
**Exon 16 : c.3422C>G, p.Pro1141Arg**
**Exon 18 : c.3842G>A, p.Gly1281Asp**
**Exon 8 : c.2332C>G, p.Arg778Gly**
**Exon 8 : c.2332C>G, p.Arg778Gly**

**Exon 4 : c.1651T>G, p.Phe551Val**
**Exon 13 : c.3059A>G, p.Lys1020Arg**
**Exon 13 : c.3059A>G, p.Lys1020Arg**
**Intron 1 : c.51+4A>T, p.?**
**Exon 18 : c.3842G>A, p.Gly1281Asp**
**Exon 2 : c.576_577insG, p.Gln193AlafsTer11**
**Exon 2 : c.1155_1157delinsC, p.Glu385AspfsTer19**
**Exon 4 : c.(1543+1_1544-1)_(1707+1_1708-1)del**
**Exon 4 : c.(1543+1_1544-1)_(1707+1_1708-1)del**
**Intron 1 : c.51+4A>T, p.?**
**Exon 13 : c.2930C>T, p.Thr977Met**
**Exon 8 : c.2332C>G, p.Arg778Gly**
**Exon 5 : c.1847G>A, p.Arg616Gln**
**Exon 13 : c.3008C>T, p.Ala1003Val**
**Exon 2 : c.314C>A, p.Ser105***
**Exon 2 : c.314C>A, p.Ser105***
**Intron 3 : c.1543+1G>T, p.?**
**Intron 5 : c.1869+2T>C, p.?**
**Intron 5 : c.1869+2T>C, p.?**
**Exon 18 : c.3809A>G, p.Asn1270Ser**
**Exon 14 : c.3207C>A, p.His1069Gln**
**Exon 14 : c.3207C>A, p.His1069Gln**
**Exon 19 : c.3955C>T, p.Arg1319***
**Exon 19 : c.3955C>T, p.Arg1319***
**Exon 10 : c.2501T>A, p.Val834Asp**

**Exon 14 : c.3207C>A, p.His1069Gln**
**Exon 14 : c.3082_3085delinsG, p.Lys1028SerfsX40**
**Exon 14 : c.3082_3085delinsG, p.Lys1028SerfsX40**
**Exon 7 : c.2007_2013del, p.Tyr670***
**Exon 14 : c.3207C>A, p.His1069Gln**
**Exon 14 : c.3207C>A, p.His1069Gln**
**Exon 14 : c.3207C>A, p.His1069Gln**
**Exon 14 : c.3207C>A, p.His1069Gln**
**Exon 14 : c.3207C>A, p.His1069Gln**
**Exon 14 : c.3207C>A, p.His1069Gln**
**Exon 14 : c.3207C>A, p.His1069Gln**
**Exon 13 : c.3007G>A, p.Ala1003Thr**
**Exon 8 : c.2128G>A, p.Gly710Ser**
**Intron 1 : c.51+4A>T, p.?**
**Exon 13 : c.3007G<A, p.Ala1003Thr and Exon14: c.3106G>A, p.Val1036Ile**
**Exon 13 : c.3007G<A, p.Ala1003Thr and Exon14: c.3106G>A, p.Val1036Ile**
**Exon 14 : c.3191A>C, p.Glu1064Ala**
**Exon 14 : c.3207C>A, p.His1069Gln**
**Exon 14 : c.3207C>A, p.His1069Gln**
**Exon 14 : c.3207C>A, p.His1069Gln**
**Exon 11 : c.2716G>C, p.Ala906Pro**
**Exon 17 : c.3659C>T, p.Thr1220Met**
**Exon 14 : c.3207C>A, p.His1069Gln**
**Exon 14 : c.3207C>A, p.His1069Gln**
**Exon 11 : c.2621C>T, p.Ala874Val**

**Exon 11 : c.2621C>T, p.Ala874Val**
**Exon 17 : c.3672_3674delinsTTCC, p.Lys1225Serfs*34**
**Exon 17 : c.3672_3674delinsTTCC, p.Lys1225Serfs*34**
**Exon 8 : c.2336G>A, p.Trp779***
**Exon 14 : c.3082_3085delinsG, p.Lys1028SerfsX40**
**Exon 21 : c.4144G>T; p.Glu1382***
**Exon 21 : c.4144G>T; p.Glu1382***
**Exon 18 : c.3809A>G, p.Asn1270Ser**
**Intron 5 : c.1869+5_1869+8delGTAA, p.?**
**Exon 8 : c.2333G>T, p.Arg778Leu**
**Exon 13 : c.2936T>A, p.Leu979Gln**
**Exon 14 : c.3104G>T, p.Gly1035Val**
**Exon 14 : c.3121C>T, p.Arg1041Trp**
**Exon 14 : c.3121C>T, p.Arg1041Trp**
**Intron 5 : c.1869+5_1869+8delGTAA, p.?**
**Intron 5 : c.1869+5_1869+8delGTAA, p.?**
**Exon 5 : c.1772G>A p.Gly591Asp**
**Exon 8 : c.2128G>A, p.Gly710Ser**
**Exon 16 : c.3461T>C, p.Leu1154Pro**
**Exon 16 : c.3461T>C, p.Leu1154Pro**
**Exon 16 : c.3461T>C, p.Leu1154Pro**
**Exon 18 : c.3842G>A, p.Gly1281Asp**
**Exon 17 : c.3617_3618insA, p.His1207Alafs*52**
**Exon 13 : c.3053C>T, p.Ala1018Val**
**Exon 8 : c.2128G>A, p.Gly710Ser**

**Exon 14 : c.3207C>A, p.His1069Gln**
**Exon 21 : c.4144G>T, p.Glu1382***
**Exon 20 : c.4092_4093delGT, p.Ser1365Lysfs*12**
**Exon 14 : c.3188C>T, p.Ala1063Val**
**Exon 14 : c.3188C>T, p.Ala1063Val**
**Exon 8 : c.2336G>A, p.Trp779***
**Exon 11 : c.2621C>T, p.Ala874Val**
**Exon 13 : c.3059A>G, p.Lys1020Arg**
**Exon 13 : c.3059A>G, p.Lys1020Arg**
**Exon 9 : c.2391_2393del, p.Leu798del**
**Exon 14 : c.3207C>A, p.His1069Gln**
**Exon 9 : c.2383C>T, p.Leu795Phe**
**Exon 9 : c.2383C>T, p.Leu795Phe**
**Exon 13 : c.3059A>G, p.Lys1020Arg**
**Intron 1 : c.51+5G>A, p.?**
**Exon 8 : c.2303C>T, p.Pro768Leu**
**Exon 13 : c.2930C>T, p.Thr977Met**
**Exon 8 : c.2304dupC, p.Met769Hisfs*26**
**Exon 14 : c.3207C>A, p.His1069Gln**
**Exon 17 : c.3694A>C, p.Thr1232Pro**
**Exon 17 : c.3694A>C, p.Thr1232Pro**
**Exon 17 : c.3694A>C, p.Thr1232Pro**
**Exon 10 : c.2519C>T, p.Pro840Leu**
**Exon 10 : c.2549C>T, p.Thr850Ile**
**Exon 10 : c.2549C>T, p.Thr850Ile**

**Exon 10 : c.2549C>T, p.Thr850Ile**
**Exon 2 : c.525dup, p.Val176SerfsX28**
**Exon 5 : c.1846C>T, p.Arg616Trp**
**Exon 5 : c.1846C>T, p.Arg616Trp**
**Exon 5 : c.1846C>T, p.Arg616Trp**
**Exons 2 - 5 : c.(51+1_52-1)_(1285+1_1286-1)del**
**Exons 2 - 5 : c.(51+1_52-1)_(1285+1_1286-1)del**
**Exon 6 : c.1934T>G, p.Met645Arg**
**Exon 2 : c.505C>T, p.Gln169Ter**
**Exon 10 : c.2532del, p.Val845Serfs*28**
**Exon 10 : c.2532del, p.Val845Serfs*28**
**Exon 10 : c.2532del, p.Val845Serfs*28**
**Intron 18 : c.3904-2A>G, p.?**
**Intron 18 : c.3904-2A>G, p.?**
**Exon 8 : c.2292C>T, p.Phe764Phe**
**Exon 14 : c.3207C>A, p.His1069Gln**
**Exon 8 : c.2332C>G, p.Arg778Gly**
**Exon 14 : c.3207C>A, p.His1069Gln**
**Exon 14 : c.3207C>A, p.His1069Gln**
**Exon 14 : c.3082_3085delinsG, p.Lys1028SerfsX40**
**Exon 5 : c.1745_1746delTA, p.Ile582Arg*25**
**Exon 4 : c.1705_1707+10del113, p.Thr569del**
**Exon 18 : c.3845dupT, p.Ala1283GlyfsX21**
**Exon 2 : c.1155_1157delinsC, p.Glu385AspfsX19**
**Exon 14 : c.3207C>A, p.His1069Gln**

**Exon 6 : c.1877G>C, p.Gly626Ala**
**Exon 13 : c.3008C>T, p.Ala1003Val**
**Exon 14 : c.3121C>T, p.Arg1041Trp**
**Exon 13 : c.3008C>T, p.Ala1003Val**
**Exon 8 : c.2333G>T, p.Arg778Leu**
**Exon 8 : c.2333G>T, p.Arg778Leu**
**Exon 8 : c.2333G>T, p.Arg778Leu**
**Exon 14 : c.3207C>A, p.His1069Gln**
**Exon 2 : c.676C>T,p.Arg226Trp and Exon 8 : c.2297C>G, p.Thr766Arg**
**Intron 7 : c.2122-8T>G, p.?**
**Exon 8 : c.2304dupC, p.Met769Hisfs*26**
**Intron 5 : c.1869+2T>C, p.?**
**Exon 16 : c.3532A>G, p.Thr1178Ala**
**Exon 14 : c.3207C>A, p.His1069Gln**
**Exon 14 : c.3207C>A, p.His1069Gln**
**Exon 14 : c.3207C>A, p.His1069Gln**
**Exon 7 : c.2046G>C, p.Met682Ile**
**Exon 2 : c.915T>A, p.Cys305***
**Exon 8 : c.2305A>G, p.Met769Val**
**Exon 8 : c.2304dupC, p.Met769Hisfs*26**
**Exon 18 : c.3818C>T, p,Pro1273Leu**
**Exon 14 : c.3207C>A, p.His1069Gln**
**Exon 8 : c.2304dupC, p.Met769Hisfs*26**
**Exon 8 : c.2304dupC, p.Met769Hisfs*26**
**Exon 3 : c.1369C>T, p.Gln457***

**Exon 3 : c.1369C>T, p.Gln457***
**Intron 2 : c.1286-416A>G, p.?**
**Intron 1 : c.51+4A>T, p.?**
**Exon 8 : c.2304C>T, p.Met 775Val**
**Exon 14 : c.3207C>A, p.His1069Gln**
**Exon 2 : c.1155_1157delinsC, p.Glu385Aspfs*19**
**Exon 4 : c.1625T>C, p.Ile542Thr**
**Intron 11 : c.2731-1G>A, p.?**
**Intron 11 : c.2731-1G>A, p.?**
**Exon 12 : c.2753A>G, p.Asp918Gly**
**Exon 16 : c.3556G>A, p.Gly1186Ser**
**Exon 10 : c.2519C>T, p.Pro840Leu**
**Exon 10 : c.2519C>T, p.Pro840Leu**
**Intron 1 : c.51+4A>T, p.?**
**Exon 3: c.1369C>T, p.Glu457***
**Intron 1 : c.51+4A>T, p.?**
**Exon 13 : c.3008C>T, p.Ala1003Val**
**Exon 2 : c.865C>T, p.Gln289***
**Intron 1 : c.51+4A>T, p.?**
**Exon 8 : c.2304dupC, p.Met769Hisfs*26**
**Exon 10 : c.2519C>T, p.Pro840Leu**
**Exon 14 : c.3207C>A, p.His1069Gln**
**Exon 14 : c.3207C>A, p.His1069Gln**
**Exon 14 : c.3207C>A, p.His1069Gln**
**Exon 16 : c.3551T>C, p.Ile1184Thr**

**Exon 16 : c.3551T>C, p.Ile1184Thr**
**Exon 7 : c.2007_2013del, p.Tyr670***
**Exon 8 : c.2305A>G, p.Met769Val**
**Exon 7 : c.2009_2015del, p.Tyr670***
**Exon 7 : c.2009_2015del, p.Tyr670***
**Exon 13 : c.2912C>T, p.Ala971Val**
**Exon 16 : c.3548C>G, p.Ala1183Gly**
**Exon 2 : c.331C>T, p.Gln111***
**Exon 14 : c.3207C>A, p.His1069Gln**
**Exon 8 : c.2305A>G, p.Met769Val**
**Intron 18 : c.3904-2A>G, p.?**
**Exon 14 : c.3207C>A, p.His1069Gln**
**Exon 14 : c.3207C>A, p.His1069Gln**
**Exon 14 : c.3207C>A, p.His1069Gln**
**Intron 1 : c.51+4A>T, p.?**
**Exon 14 : c.3207C>A, p.His1069Gln**
**Exon 8 : c.2304dupC, p.Met769Hisfs*26**
**Exon 14 : c.3207C>A, p.His1069Gln**
**Exon 14 : c.3207C>A, p.His1069Gln**
**Exon 14 : c.3207C>A, p.His1069Gln**
**Exon 18 : c.3818C>A, p.Pro1273Gln**
**Exon 18 : c.3842G>A, p.Gly1281Asp**
**Exon 6 : c.1877G>C, p.Gly626Ala**
**Exon 13 : c.3007G>C, p.Ala1003Pro**
**Exon 14 : c.3207C>A, p.His1069Gln**

**Exon 14 : c.3207C>A, p.His1069Gln**
**Exon 14 : c.3207C>A, p.His1069Gln**
**Exon 14 : c.3207C>A, p.His1069Gln**
**Exon 14 : c.3207C>A, p.His1069Gln**
**Exon 14 : c.3207C>A, p.His1069Gln**
**Exon 14 : c.3207C>A, p.His1069Gln**
**Exon 14 : c.3207C>A, p.His1069Gln**
**Exon 14 : c.3207C>A, p.His1069Gln**
**Exon 14 : c.3207C>A, p.His1069Gln**
**Exon 14 : c.3207C>A, p.His1069Gln**

		Number of values	Mean	Median	Minimum	Maximum	2.5% Percentile	97.5% Percentile	p-value
**Total serum copper (µmol/L)**	F	126	17.5	16.6	7.6	35.4	10.5	31.7	0.0098
	M	89	15.8	14.9	2.9	28.4	7.8	26.3	
**Exchangeable Copper (CuEXC) (µmol/L)**	F	126	0.88	0.86	0.45	1.90	0.48	1.45	0.4099
	M	89	0.84	0.84	0.49	1.38	0.52	1.29	
**Relative Exchangeable copper (REC) (%)**	F	126	5.23	5.00	2.20	12.80	2.62	9.77	0.0537
	M	89	5.81	5.40	2.40	19.10	2.50	9.30	
**Ceruloplasmin (g/L)**	F	117	0.276	0.270	0.170	0.479	0.190	0.410	0.0083
	M	80	0.251	0.239	0.090	0.390	0.142	0.390	
**Urinary Copper (µmol/24h)**	F	51	0.19	0.15	0.02	0.97	0.02	0.87	0.013
	M	41	0.33	0.19	0.08	2.07	0.08	2.07	
**Age (years)**	F	126	28	27	2	77	3	64	0.0719
	M	89	24	21	2	72	3	66	
**AST (IU/L)**	F	99	25	22	15	52	16	41	< 0.0001
	M	75	33	29	14	200	16	118	
**ALT (IU/L)**	F	99	19	18	6	69	9	35	0.0114
	M	75	30	20	6	256	8	256	

Table S2. Results of copper metabolism assessment in controls, with comparisons by sex

Serum copper and CuEXC conversion: µmol/L × 6.3546 = µg/dL. F: female; M: male;

		Number of values	Mean	Median	Minimum	Maximum	2.5% Percentile	97.5% Percentile	p-value
**Age (years)**	<16 y	72	7	7	2	15	2	15	
	≥16 y	143	36	35	16	77	16	69	
**Total serum copper (µmol/L)**	<16 y	72	17	17	8	28	9	27	0.1338
	≥16 y	143	17	16	3	35	8	30	
**Exchangeable Copper (CuEXC) (µmol/L)**	<16 y	72	0.79	0.77	0.48	1.32	0.50	1.21	0.0006
	≥16 y	143	0.90	0.88	0.45	1.90	0.53	1.41	
**Relative Exchangeable copper (REC) (%)**	<16 y	72	4.91	4.70	2.40	12.80	2.48	10.74	0.0004
	≥16 y	143	5.75	5.40	2.20	19.10	3.26	9.42	
**Ceruloplasmin (g/L)**	<16 y	65	0.278	0.280	0.170	0.400	0.195	0.394	0.0243
	≥16 y	132	0.259	0.250	0.090	0.479	0.145	0.406	
**Urinary Copper (µmol/24h)**	<16 y	35	0.24	0.14	0.05	2.07	0.05	2.07	0.1344
	≥16 y	57	0.26	0.18	0.02	2.02	0.02	1.46	
**AST (IU/L)**	<16 y	60	32	33	16	55	17	53	< 0.0001
	≥16 y	114	26	22	14	200	16	109	
**ALT**	<16 y	60	17	18	6	31	6	28	0.0035

**(IU/L)**	≥16 y	114	27	20	9	256	11	163	

Table S3. Results of copper metabolism assessment in controls, with comparisons across age groups.

Serum copper and CuEXC conversion: µmol/L × 6.3546 = µg/dL. F: female; M: male.

Group	Gender	Age	Variant 1	Variant 2	Phenotype	Total Cu[1] (µmol/L)	Total Cu[1] (µg/dL)	CuEXC[2] (µmol/L)	CuEXC[2] (µg/dL)	REC[3] (%)	Cp[4] (g/L)	24 hr-UCE[5] (µmol/24h)	AST (U/L)	ALT (U/L)	PT (%)	Platelet count (G/L)	Hemoglobin	KFR	
WD	F	30	c.3007G>A	c.3106G>A	Hepatic	10.61	67.4	1.37	8.7	12.91	0.16	NA	52	55	71	76	12.6	Yes	False negative
WD	F	1	c.1708-1G>A	c.3620A>G. p.His1207Arg	Asymptomatic	20.55	130.6	0.75	4.8	3.65	0.47	NA	42	33	100	233	11.3	NA	False negative
WD	M	13	c.1708-1G>A	c.1708-1G>A	Hepatic	11.50	73.1	1.05	6.7	9.13	0.18	NA	43	88	91	221	15.8	No	False negative
WD	F	8	c.2128G>A. p.Gly710Ser	c.3505A>G. p.Met1169Val	Hepatic	13.66	86.8	1.3	8.3	9.52	0.22	1.57	56	80	88	290	14.1	No	False negative
WD	M	53	c.2804C>T p.Thr935Met	c.2804C>T p.Thr935Met	Asymptomatic	10.00	63.5	1.15	7.3	11.50	0.22	0.39	28	31	93	267	14.5	No	False negative
WD	F	35	c.2804C>T p.Thr935Met	c.2804C>T p.Thr935Met	Asymptomatic	14.00	89	0.97	6.2	6.93	0.28	0.33	20	18	87	257	13.9	No	False negative
WD	F	12	c.1708-1G>A	c.1708-1G>A	Hepatic	12.47	79.2	1.4	8.9	11.23	0.18	2.88	196	336	88	328	11.8	No	False negative
WD	F	21	c.670dup p.Ile224Asnfs*2	c.2046G>C. p.Met682Ile	Asymptomatic	15.20	96.6	0.89	5.7	5.86	0.22	0.32	21	16	100	280	13.9	No	False negative
WD	M	8	c.2128G>A p.gLY710Ser	c.2305A>G. p.Met769Val	Hepatic	15.79	100.3	2.10	13.3	13.30	0.25	2.7	105	253	91	346	13.2	No	False negative
Control	M	21	NA	NA	CDG	2.88	18.3	0.55	3.5	19.1	0.09	0.5	200	150	100	NA	NA	NA	False positive

																		(CDG diagnosis)

Table S4. Main clinical, biological and genetic features associated with false negative and false positive REC results.

F: Female; M: Male; Total Cu: Total serum copper; CuEXC: Exchangeable copper; REC: Relative exchangeable copper; Cp: Ceruloplasmin; 24 hr-UCE: 24-hour urinary copper excretion; AST: Aspartate aminotransferase; ALT: Alanine aminotransferase; PT: Prothrombin rate; KFR: Kayser Fleischer Ring; CDG: Congenital Disorder of Glycosylation.

Reference ranges were determined across all control samples

[1] Reference range: 8.5-28.2 µmol/L (24-179.2 µg/dL)

[2] Reference range: 0.50-1.38 µmol/L (3.2-8.8 µg/dL)

[3] Reference range: 2.6-9.5%

[4] Reference range: 0.17-0.4 g/L

[5] Reference range: 0.03-1.679 µmol/24h

Table S5. Comparison of total copper, exchangeable copper (CuEXC), and relative exchangeable copper (REC) levels in controls and heterozygous carriers (HTZ) across two time periods (2009–2014 vs. after 2015).

		Period	n	Median	Mean	Std. Deviation	P value
Total copper (µmol/L)	Controls	2009-2014	59	16,2	16,24	3,356	0,895
		After 2015	102	15,75	16,52	4,309	
	HTZ	2009-2014	147	14,9	15,52	4,277	0,0983
		After 2015	123	14,01	14,74	4,128	
Exchangeable copper (µmol/L)	Controls	2009-2014	59	0,91	0,92	0,18	0,0218
		After 2015	102	0,83	0,85	0,21	
	HTZ	2009-2014	147	0,88	0,90	0,20	0,5336
		After 2015	121	0,89	0,94	0,29	
Relative exchangeable copper (%)	Controls	2009-2014	59	5,5	5,84	1,52	0,0889
		After 2015	102	5,1	5,46	1,93	
	HTZ	2009-2014	147	5,9	6,10	1,78	0,0508
		After 2015	123	6,3	6,68	2,52	
